# Research on volatile flavor substances and biological activities of different parts of *Allium schoenoprasum* L.

**DOI:** 10.3389/fnut.2026.1842096

**Published:** 2026-06-18

**Authors:** Chuanshun Zhou, Lisha Yan, Ming Cheng, Min Tang

**Affiliations:** 1 Department of Pharmacy, Yiyang Medical College, Yiyang, China; 2 Department of Clinical Medicine, Yiyang Medical College, Yiyang, China

**Keywords:** *Allium schoenoprasum* L., cytokine-modulating activity, GC–IMS, radical-scavenging activity, volatile fingerprints

## Abstract

**Objective:**

To systematically characterize the differences in volatile flavor compounds among the fibrous roots, white shafts, and green leaves of chives (*Allium schoenoprasum* L.), and to evaluate the preliminary *in vitro* antioxidant and cytokine-modulating activities of their corresponding essential oil fractions, thereby providing a comparative basis for the differentiated evaluation of chive-derived materials.

**Methods:**

Volatile compounds in different chive parts were systematically analyzed using GC–IMS combined with PCA and PLS–DA. The antioxidant activities of the essential oils were evaluated by ABTS and DPPH radical scavenging assays. Their cytokine-modulating effects were assessed in an LPS-induced RAW264.7 macrophage model by measuring TNF-α, IL-6, and IL-1β levels.

**Results:**

A total of 175 volatile signals were detected and tentatively annotated. Clear differences were observed among the three chive tissues in terms of compound composition, relative abundance, and overall volatile fingerprints. Fibrous roots contained a broader diversity of volatile signals and showed a relatively higher abundance of aldehydes; white shafts were characterized by relatively abundant ester compounds; and green leaves showed a higher proportion of sulfur-containing compounds. PCA and PLS–DA separated the three tissue types, indicating a tissue-dependent pattern in the volatile characteristics of chives. Based on EC50 values, fibrous root essential oil showed stronger radical-scavenging activity in both ABTS and DPPH assays than white shaft and green leaf oils. In the LPS-induced RAW264.7 macrophage model, the three essential oil fractions reduced TNF-α, IL-6, and IL-1β release to varying degrees at the tested concentration.

**Conclusion:**

Different parts of chives exhibited distinct volatile profiles and preliminary *in vitro* bioactivity patterns. The results indicate that anatomical tissue type is an important factor influencing the volatile characteristics and activity profiles of chive-derived materials. This study provides a comparative basis for the differentiated evaluation and further targeted investigation of fibrous roots, white shafts, and green leaves of *Allium schoenoprasum* L.

## 1 Introduction

Chives (*Allium schoenoprasum* L.) are widely used as culinary herbs because of their characteristic aroma and potential health-promoting properties (1–3). Similar to other *Allium* vegetables, the sensory quality and reported bioactivity-related properties of chives are closely associated with their volatile constituents, particularly sulfur-containing compounds, aldehydes, alcohols, esters, and terpenoids (4–6). These compounds are major contributors to the typical flavor profile of chives and may also be associated with reported bioactivities of Allium-derived materials, which are relevant to food quality evaluation, natural preservation, and preliminary bioactivity-oriented assessment (7–10).

Considerable attention has been paid to the volatile composition and bioactivities of *Allium* species such as onion, garlic, and chive (11). In Allium plants, organosulfur compounds, aldehydes, alcohols, esters, and terpenoids are not only important contributors to characteristic aroma, but may also provide chemical background for evaluating tissue-related differences in preliminary *in vitro* bioactivity (11, 12). IBecause these volatile constituents are often present as complex mixtures and may vary among plant tissues, a rapid fingerprinting approach is useful for comparing their distribution patterns. Gas chromatography–ion mobility spectrometry (GC–IMS) has been increasingly applied to the rapid visualization and discrimination of food volatiles, making it suitable for comparing tissue-dependent volatile fingerprints in plant-derived aromatic materials. Therefore, GC–IMS was used in the present study to characterize the volatile differences among fibrous roots, white shafts, and green leaves of chives, while subsequent *in vitro* assays were performed to preliminarily compare the activity patterns of their corresponding essential oil fractions.

For chives, fibrous roots, white shafts, and green leaves differ substantially in physiological function and environmental exposure, which may lead to distinct volatile profiles and tissue-associated activity patterns (13, 14). In addition, studies on Chinese chive and related *Allium* species have reported antimicrobial or anti-inflammatory activities of volatile-rich extracts and sulfur-containing constituents, suggesting the value of combining volatile profiling with preliminary *in vitro* bioactivity evaluation (15, 16). Beyond their contribution to aroma, volatile and semi-volatile constituents in *Allium* plants may provide chemical background for preliminary *in vitro* bioactivity evaluation. Since different plant tissues may accumulate different volatile fractions, it is reasonable to compare whether these tissue-associated differences are accompanied by distinct radical-scavenging and cytokine-modulating patterns. Therefore, ABTS (2,2′-azino-bis(3-ethylbenzothiazoline-6-sulfonic acid) and DPPH (2,2-diphenyl-1-picrylhydrazyl)) assays were used as chemical radical-scavenging models, and the LPS-induced RAW264.7 macrophage model was used to assess cytokine-related responses. Nevertheless, most available studies have focused on whole plants, general *Allium* species, or processed products, whereas systematic comparisons among different anatomical parts of *Allium schoenoprasum* L. remain limited.

**Figure 1:**
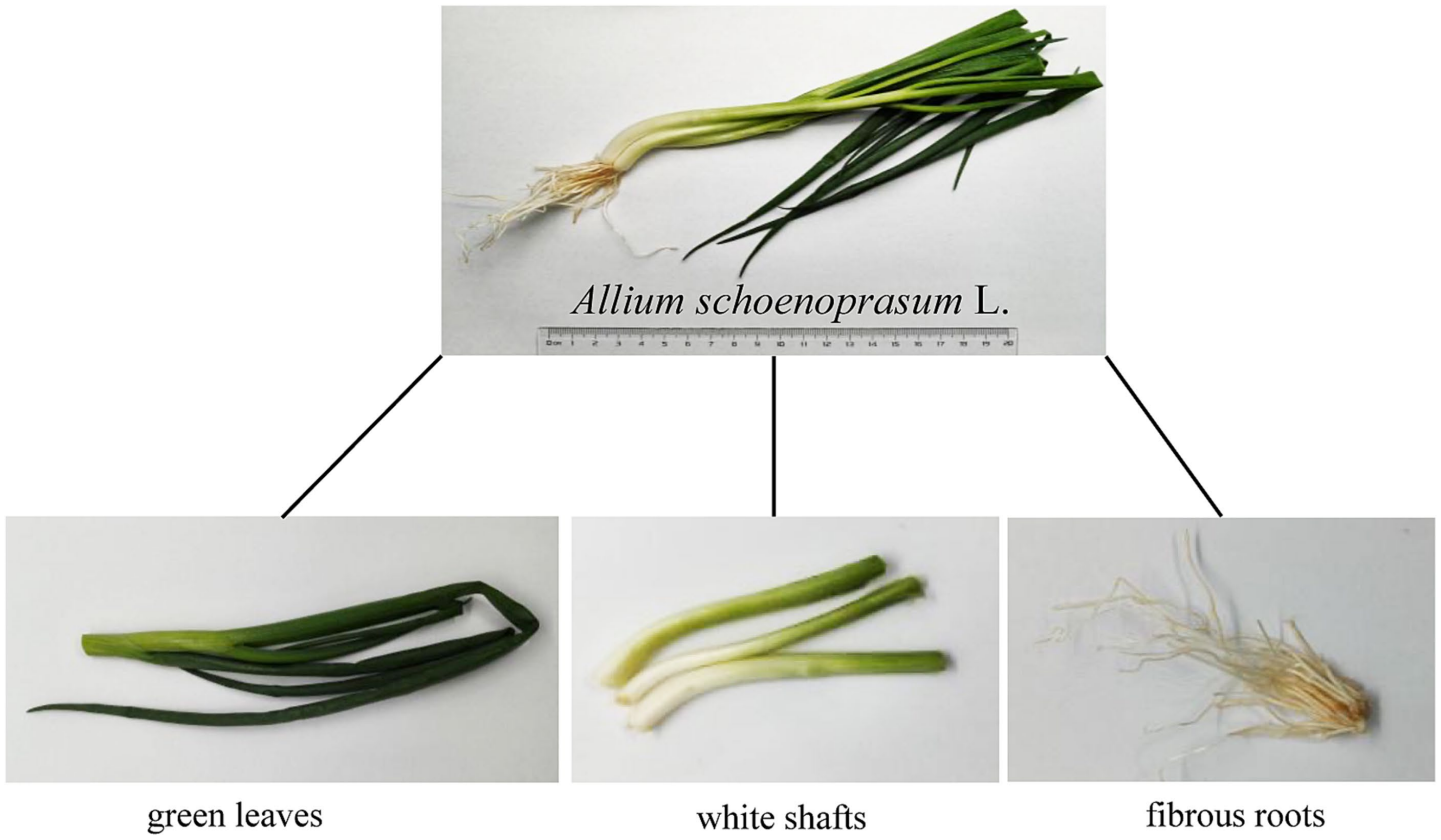
Fibrous roots, white shafts, and green leaves of chives (*Allium schoenoprasum* L.).

Therefore, this study aimed to characterize the tissue-specific volatile fingerprints of fibrous roots, white shafts, and green leaves of chives (Figure 1) using GC–IMS, and to further evaluate the preliminary *in vitro* radical-scavenging and cytokine-modulating activities of the corresponding essential oil fractions. This study provides a comparative basis for understanding tissue-associated volatile differences and preliminary *in vitro* activity patterns of chive-derived materials.

## 2 Materials and methods

### 2.1 Materials, reagents, microorganisms, and instruments

Nitrogen gas (99.999%) was purchased from Yiyang Zhongda Gas Co., Ltd. (Yiyang, China). Twenty-milliliter headspace vials were obtained from Hanon Instruments Co., Ltd. (Shandong, China). A DK-3001A headspace sampler was purchased from Zhongxing Analysis Instrument New Technology Research Institute (China). A GC-2030 gas chromatograph equipped with an SH-WAX capillary column (30 m × 0.32 mm × 0.25 μm) was obtained from Shimadzu Corporation (Kyoto, Japan). An IMS-S ion mobility spectrometer was purchased from Gesellschaft für Analytische Sensorsysteme mbH (G. A. S., Dortmund, Germany). Dimethyl sulfoxide (DMSO) and 96-well microplates were purchased from Shanghai Yuanye Bio-Technology Co., Ltd. (Shanghai, China). A thermostatic shaking incubator (YP-TY1) was purchased from Shandong Youyunpu Optoelectronic Technology Co., Ltd. (Shandong, China). A microplate reader (SpectraMax iD3) was obtained from Molecular Devices (San Jose, CA, United States). RAW264.7 cells (CL-0190), high-glucose Dulbecco’s modified Eagle’s medium (DMEM; PM150213A), Fetal Bovine Serum (164210), and penicillin–streptomycin solution (PB180120) were obtained from Procell Life Science & Technology Co., Ltd. (Wuhan, China). Lipopolysaccharide (LPS, L2880) was purchased from Sigma-Aldrich (St. Louis, MO, United States). Cell Counting Kit-8 (CCK-8) (HY-K0301) was obtained from MedChemExpress (MCE, Monmouth Junction, NJ, United States). A CO_2_ incubator (Steri-Cycle CO_2_) was purchased from Thermo Scientific (Waltham, MA, United States). Enzyme-linked immunosorbent assay (ELISA) kits for tumor necrosis factor-α (TNF-α) (CSB-E04741m), interleukin-6 (IL-6) (CSB-E04639m), and interleukin-1β (IL-1β) (CSB-E08054m-MS) were purchased from Cusabio Biotech Co., Ltd. (Wuhan, China). ABTS Total Antioxidant Capacity Assay Kit (catalog no. R20223) and the DPPH Radical Scavenging Activity Assay Kit (catalog no. R27137-100 T) was purchased from Shanghai Yuanye Bio-Technology Co., Ltd. (Shanghai, China). L-ascorbic acid (vitamin C; catalog no. ST1434-25 g) were purchased from Beyotime Biotechnology Co., Ltd. (Shanghai, China).

### 2.2 Plant materials and sample preparation

Fresh *Allium schoenoprasum* L. samples were purchased on October 8, 2025, from Yiyang, Hunan Province, China. The chives were originally cultivated in Huarong County, Yueyang City, Hunan Province, China, under conventional open-field cultivation conditions. According to the supplier, the samples were harvested within 1–2 days prior to purchase during the regular seasonal harvesting period. All samples were collected from the same production area, at the same growth stage, and on the same harvesting date to minimize variability associated with plant maturity, cultivation background, and post-harvest handling. After purchase, the fresh plant materials were transported to the laboratory under clean conditions and processed immediately upon arrival. Because detailed cultivation parameters such as altitude, soil physicochemical properties, fertilization records, and microenvironmental conditions were not available from the supplier, the present study mainly reflects the volatile composition characteristics and preliminary *in vitro* activity patterns of commercially available representative chive samples. Potential variations associated with different geographical origins, cultivation conditions, and harvesting seasons should be further evaluated in future studies.

Fresh chives (*Allium schoenoprasum* L.) were identified by Professor Dan Huang, Hunan University of Chinese Medicine. The fresh chives were manually separated into fibrous roots, white shafts, and green leaves. The fresh chives were manually separated into fibrous roots, white shafts, and green leaves according to visible anatomical position and tissue color. Fibrous roots were defined as the root portion below the basal plate. White shafts were defined as the basal white to pale-green pseudostem region, whereas green leaves were defined as the upper visibly green leaf blade region. During separation, transitional tissues between the white shafts and green leaves were minimized as much as possible to reduce cross-contamination among anatomical parts. Each part was cut into pieces of approximately 1.0–2.0 mm. Then, 2.0000 ± 0.0005 g of each sample was accurately weighed into a 20 mL headspace vial. Three parallel replicates were prepared for each sample.

### 2.3 GC–IMS analysis of volatile compounds

#### 2.3.1 Headspace sampling conditions

The headspace vials were incubated at 90 °C for 15 min. The injection volume was 1 mL. The syringe temperature was set at 95 °C, and the injector needle temperature was set at 100 °C.

#### 2.3.2 Gas chromatographic conditions

Volatile compounds were separated on an SH-WAX capillary column (30 m × 0.32 mm × 0.25 μm) using high-purity nitrogen (≥99.999%) as the carrier gas. The initial column temperature was 45 °C and maintained for 2.0 min, then increased to 150 °C at a rate of 6 °C/min and held for 10.0 min. The total run time was 29.5 min. The injector temperature was 250 °C, and the split ratio was 1:1.

#### 2.3.3 Ion mobility spectrometry conditions

The IMS detector was operated with a tritium source (^3^H) in positive ion mode. The electric field strength was 500 V/cm. The drift tube length was 53 mm, and the drift tube temperature was maintained at 45 °C. High-purity nitrogen (≥99.999%) was used as the drift gas.

### 2.4 Extraction of essential oils

Fibrous roots, white shafts, and green leaves were separately weighed (500 g each) and transferred into round-bottom flasks. Distilled water (3 L) and 150 mL of 5% sodium chloride aqueous solution were added, and the samples were subjected to steam distillation for 5 h. The distillates were extracted with diethyl ether, and the organic phases were transferred to constant-weight flasks. After removal of the solvent by rotary evaporation, the essential oils were obtained. The extraction yields of essential oils were 0.0026% for fibrous roots, 0.0058% for white shafts, and 0.0022% for green leaves.

### 2.5 Determination of antioxidant activity

#### 2.5.1 ABTS radical scavenging assay

The ABTS radical scavenging activity of essential oils from different chive parts was determined according to the instructions of the commercial kit, with minor modifications (17). Briefly, the ABTS stock solution was prepared by mixing 7 mmol/L ABTS with 2.45 mmol/L potassium persulfate and allowing the mixture to react in the dark for 12–16 h at room temperature. Before use, the stock solution was diluted with absolute ethanol to obtain the ABTS working solution, and the absorbance was adjusted to 0.70 ± 0.02 at 734 nm. Essential oils from fibrous roots, white shafts, and green leaves were dissolved and diluted to final concentrations of 100, 200, 400, 600, 800, and 1,000 μg/mL. L-ascorbic acid (VC) dissolved in absolute ethanol was used as the positive control. For each assay, 0.4 mL of sample solution was mixed with 3.6 mL of ABTS working solution. The blank control and positive control were prepared in parallel under the same conditions. After gentle mixing, all reaction mixtures were incubated at room temperature for 6–10 min, and the absorbance was measured at 734 nm. Each sample was tested in triplicate. The ABTS radical scavenging activity was calculated, and the EC50 value was obtained from the logarithmic dose–response curve to evaluate antioxidant capacity (18, 19).

#### 2.5.2 DPPH radical scavenging assay

The DPPH radical scavenging activity was determined with minor modifications based on previously reported methods. DPPH was dissolved in DMSO to prepare a 0.1 mg/mL solution. Essential oils from different chive parts were diluted to concentrations of 100, 200, 400, 600, 800, and 1,000 μg/mL. L-ascorbic acid (VC) was used as the positive control. For each assay, 100 μL of sample solution was mixed with 100 μL of DPPH solution in a 96-well plate and incubated in the dark for 30 min at room temperature. The absorbance of the reaction mixture was then measured at 515 nm and recorded as A1. Equal volumes of DMSO were used to replace the DPPH solution and the sample solution, and the corresponding absorbance values were recorded as A2 and A0, respectively. Each sample was analyzed in triplicate. The DPPH radical scavenging rate was calculated using the following equation: Scavenging rate (%) = [1 − (A1 − A2) / A0] × 100. The EC50 value was calculated from the dose–response curve and used to compare the radical scavenging capacity of the different essential oils (20–22).

### 2.6 Evaluation of anti-inflammatory activity

#### 2.6.1 Effects of essential oils on RAW264.7 cell viability

RAW264.7 cells were cultured in complete DMEM consisting of 89% high-glucose DMEM, 10% fetal bovine serum, and 1% penicillin–streptomycin solution. Cells in the logarithmic growth phase were seeded into 96-well plates at a density of 1 × 10^4^ cells/well and incubated for 24 h at 37 °C in a humidified atmosphere containing 5% CO2. After cell attachment, the culture supernatant was discarded, and the cells were divided into the following groups, with six replicate wells per group: control group, treated with 100 μL of blank medium; model group, treated with 100 μL of LPS (1 μg/mL); and treatment groups treated with essential oil-containing medium at 100, 200, 300, 400, or 500 μg/mL. The samples were dissolved in DMSO, and the final DMSO concentration was maintained at 0.1% (v/v).

After treatment for 24 h, 10 μL of CCK-8 solution was added to each well, and the cells were further incubated for 2 h. The absorbance was measured at 450 nm using a microplate reader.

#### 2.6.2 Effects of essential oil fractions on LPS-induced cytokine release in RAW264.7 cells

RAW264.7 cells were seeded into 24-well plates at a density of 3 × 10^5^ cells/well. Three replicate wells were prepared for each group. After incubation at 37 °C in a humidified 5% CO2 atmosphere for 24 h, the culture supernatant was removed. The cells were then divided into the following groups: control group, treated with 500 μL of blank medium; model group, treated with 500 μL of LPS (1 μg/mL); and treatment groups, treated with 500 μL of essential oil-containing medium (200 μg/mL), with the final concentration of LPS adjusted to 1 μg/mL.

After 24 h of incubation, the cell culture supernatants were collected and centrifuged at 4 °C and 5,000 rpm for 10 min. The supernatants were collected, and the levels of TNF-α, IL-6, and IL-1β were determined using ELISA kits according to the manufacturers’ instructions.

### 2.7 Data processing

GC–IMS data were qualitatively analyzed using the NIST 2020 database and the IMS drift time database in VOCal software. Principal component analysis (PCA) and partial least squares-discriminant analysis (PLS-DA) were performed using SIMCA 14.1 software. Heatmaps were generated using the ChiPlot online platform.

### 2.8 Statistical analysis

All data are presented as the mean ± standard deviation (SD). Statistical analyses were performed using SPSS 28.0.1.1, and graphs were generated using GraphPad Prism 8.3.0. Comparisons between two groups were generally performed using an independent-samples *t*-test, while comparisons among three or more groups were analyzed by one-way analysis of variance (ANOVA) followed by Tukey’s multiple comparison test. Differences were considered statistically significant at *p* < 0.05.

## 3 Results

### 3.1 Volatile compound profiles of different chive parts

#### 3.1.1 GC–IMS two-dimensional spectral analysis

**Figure 2:**
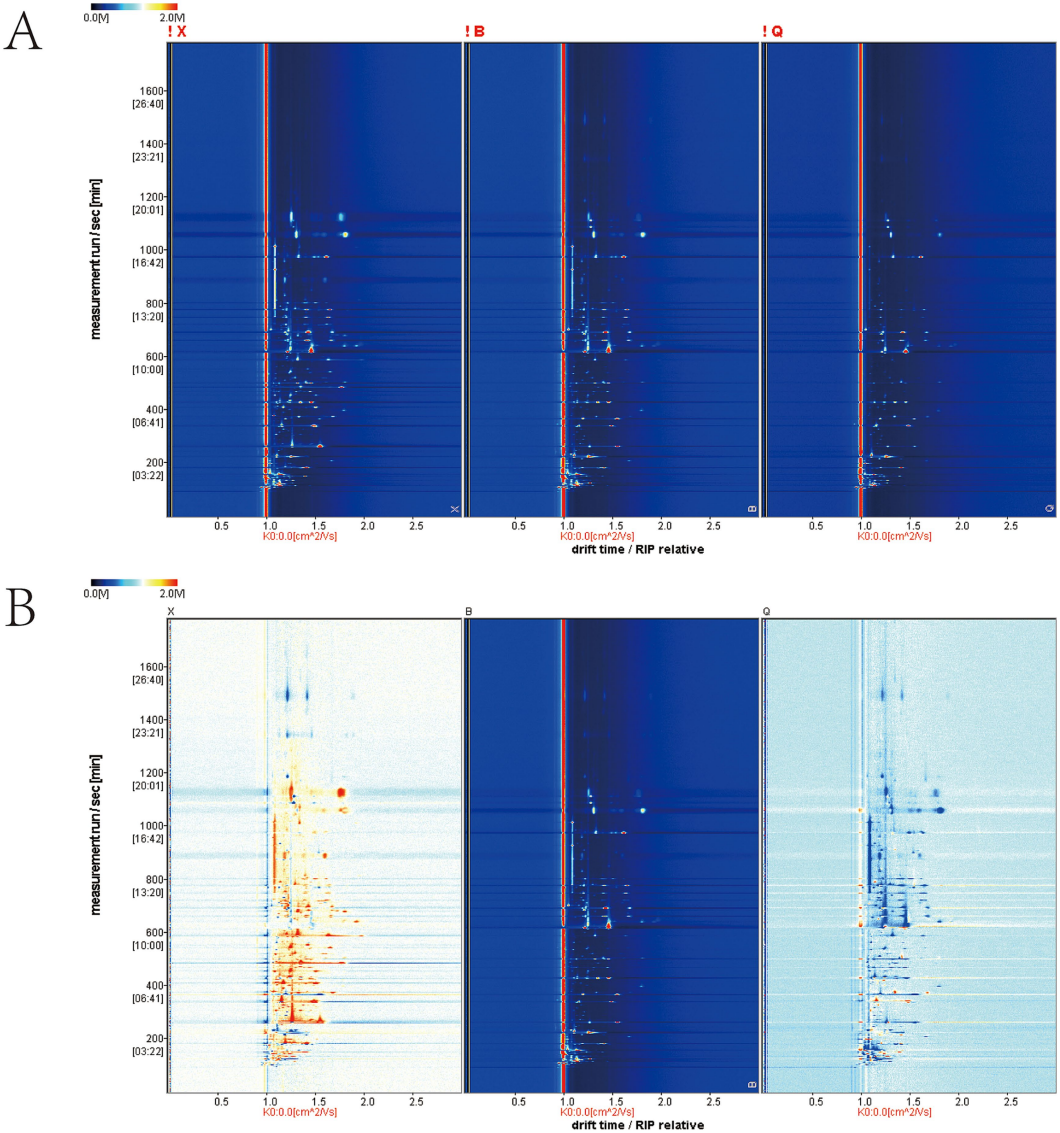
GC-IMS spectra of volatile components in the three parts (X: fibrous roots; B: white shafts; Q: green leaves) of *Allium schoenoprasum* L. (**A**: 2D spectrum; **B**: 2D difference spectrum).

GC–IMS was used to analyze the fibrous roots, white shafts, and green leaves of chives, and the spectra were processed using VOCal software. The two-dimensional spectra generated by the Reporter module showed that all three parts contained multiple volatile flavor compounds, and obvious differences in spot number and signal intensity were observed among the samples (Figure 2A). Overall, the fibrous roots showed the highest number of signal spots, followed by the white shafts, whereas the green leaves showed the fewest, indicating that fibrous roots showed a greater number and higher overall intensity of volatile signals than the other two parts.

Using the white shafts as the reference, two-dimensional difference spectra were generated (Figure 2B). The results showed that the fibrous roots were dominated by red spots, whereas the green leaves were dominated by blue spots, further indicating that the fibrous roots contained relatively higher levels of volatile compounds, while the green leaves contained fewer volatile compounds at lower relative levels.

#### 3.1.2 Qualitative analysis of volatile compounds

**Figure 3:**
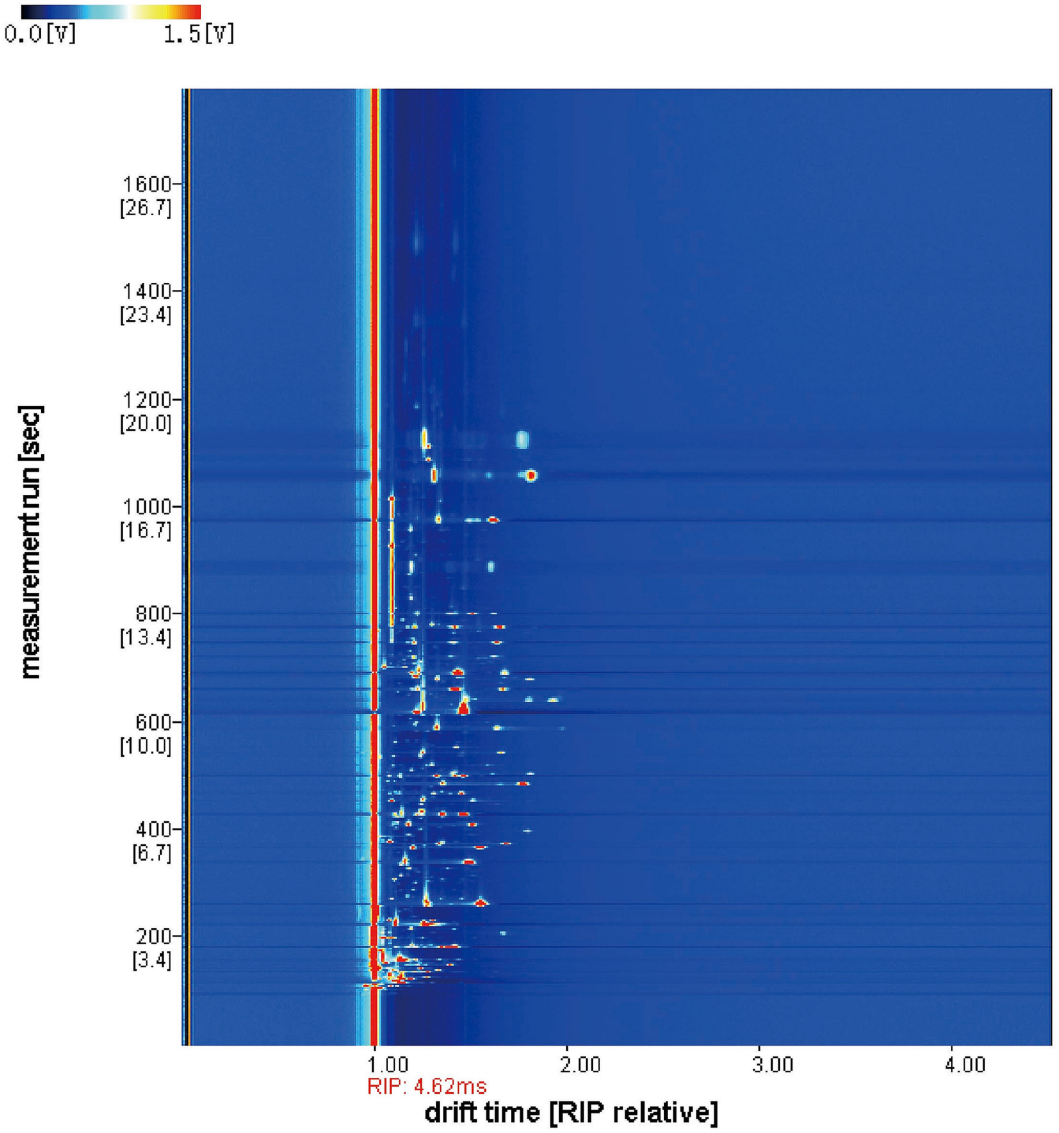
2D spectrum for qualitative analysis of volatile compounds.

**Figure 4:**
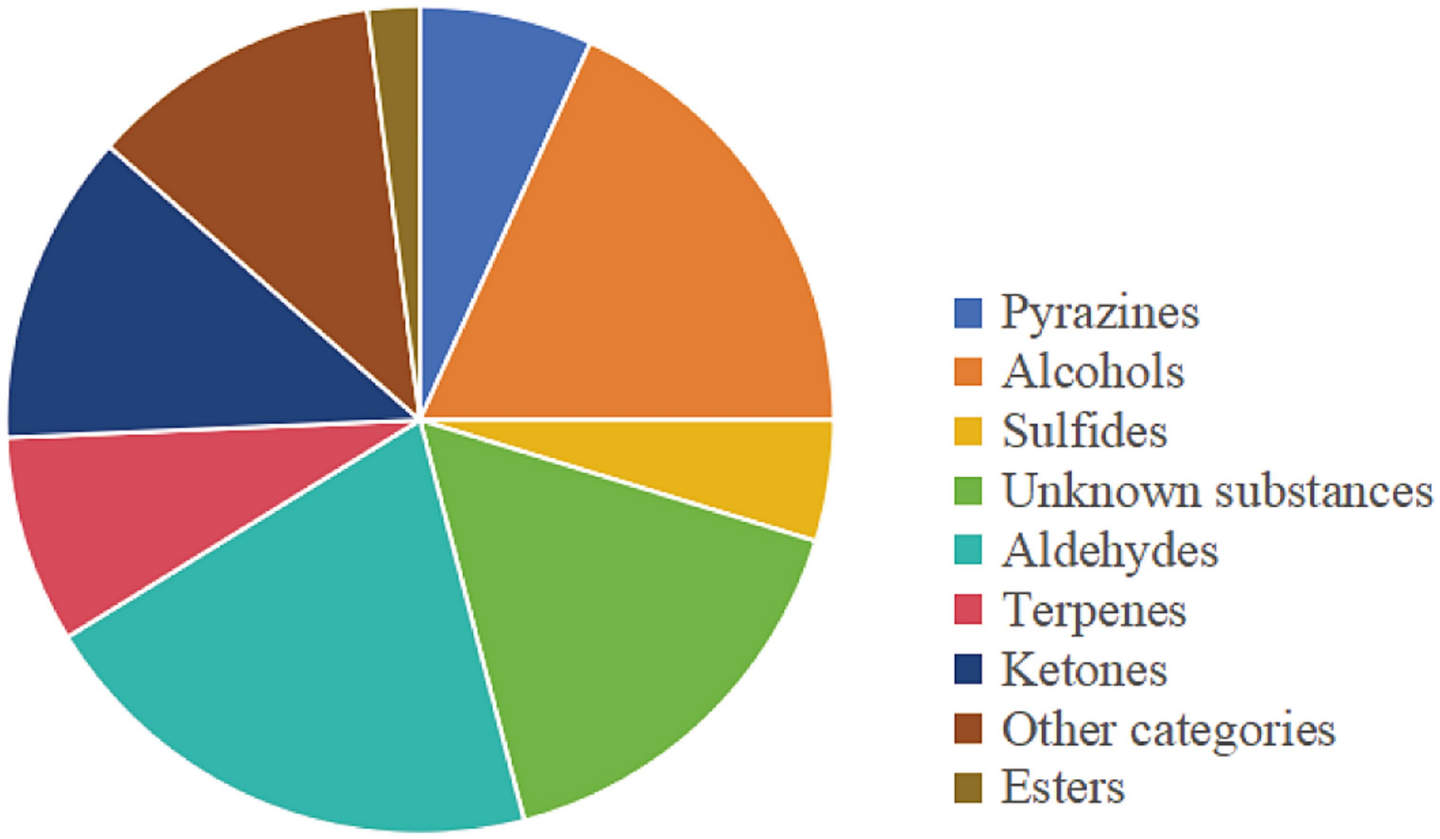
Pie chart showing the numbers of different classes of volatile flavor compounds in chives.

The spectra of the three chive parts were superimposed using the Plugin_variance module in VOCal software, and qualitative analysis was performed based on the NIST 2020 database and the IMS drift time database (Figure 3). A total of 175 volatile signals, including monomers and dimers, were detected and tentatively annotated (Table 1). These tentatively annotated signals included 30 aldehydes, 30 esters, 27 alcohols, 18 ketones, 12 terpenes, 10 pyrazines, 7 sulfur-containing compounds, 24 compounds of other classes, and 17 unknown compounds (Figure 4).

**Table 1:** Tentatively annotated volatile signals in the three parts of *Allium schoenoprasum* L.

NO	Type	Compound	CAS	Formula	MW	RI	Rt [sec]	Dt [a.u.]	Relative content %		
									Fibrous roots	White shafts	Green leaves
1	Aldehydes	Benzaldehyde	100-52-7	C_7_H_6_O	106.1	1510.0	791.145	1.14859	0.68 ± 0.10^c^	0.16 ± 0.01^a^	0.48 ± 0.03^b^
2		Methional	3268-49-3	C_4_H_8_OS	104.2	1480.4	751.646	1.08920	0.93 ± 0.12^b^	0.81 ± 0.06^b^	0.22 ± 0.03^a^
3		2,4-Heptadienal	5910-85-0	C_7_H_10_O	110.2	1465.7	732.129	1.18332	0.06 ± 0.01^a^	0.41 ± 0.03^b^	0.11 ± 0.02^a^
4		(E, E)-2,4-Nonadienal	5910-87-2	C_9_H1_4_O	138.2	1676.1	1012.648	1.34518	0.32 ± 0.00^b^	0.11 ± 0.02^a^	0.11 ± 0.03^a^
5		Phenylacetaldehyde	122-78-1	C_8_H_8_O	120.2	1649.1	976.650	1.54148	0.55 ± 0.02^a^	0.52 ± 0.05^a^	0.45 ± 0.04^a^
6		(E)-2-Nonenal	18829-56-6	C_9_H_16_O	140.2	1528.1	815.322	1.40022	0.24 ± 0.00^c^	0.10 ± 0.02^b^	0.06 ± 0.01^a^
7		Decanal	112-31-2	C_10_H_20_O	156.3	1494.3	770.243	1.54449	0.03 ± 0.00^a^	0.21 ± 0.05^b^	0.04 ± 0.00^a^
8		2-Furaldehyde	98-01-1	C_5_H_4_O_2_	96.1	1460.7	725.392	1.08089	0.25 ± 0.03^b^	0.20 ± 0.02^b^	0.08 ± 0.00^a^
9		(E)-2-Octenal M	2548-87-0	C_8_H_14_O	126.2	1427.1	680.667	1.32905	0.98 ± 0.08^c^	0.25 ± 0.05^b^	0.09 ± 0.02^a^
10		(E)-2-Octenal D	2548-87-0	C_8_H_14_O	126.2	1426.5	679.901	1.80784	0.68 ± 0.11^b^	0.07 ± 0.01^a^	0.05 ± 0.02^a^
11		Ethyl Vanillin	121-32-4	C_9_H_10_O_3_	166.2	1428.5	682.449	1.73259	0.14 ± 0.03^b^	0.04 ± 0.00^a^	0.04 ± 0.01^a^
12		1-Nonanal	124-19-6	C_9_H_18_O	142.2	1397.2	640.835	1.92922	0.86 ± 0.22^b^	0.30 ± 0.10^a^	0.31 ± 0.00^a^
13		3-Methoxy-4-hydroxybenzaldehyde	121-33-5	C_8_H_8_O_3_	152.1	1397.1	640.655	1.80469	0.49 ± 0.15^a^	0.51 ± 0.07^a^	0.26 ± 0.06^a^
14		(E)-2-Heptenal M	18829-55-5	C_7_H_12_O	112.2	1325.2	544.740	1.25220	1.26 ± 0.10^c^	0.42 ± 0.09^b^	0.17 ± 0.02^a^
15		(E)-2-Heptenal D	18829-55-5	C_7_H_12_O	112.2	1324.4	543.665	1.66477	0.84 ± 0.18^b^	0.08 ± 0.03^a^	0.05 ± 0.00^a^
16		1-Octanal D	124-13-0	C_8_H_16_O	128.2	1295.4	505.061	1.81253	0.69 ± 0.15^b^	0.35 ± 0.10^a^	0.38 ± 0.02^a^
17		1-Octanal M	124-13-0	C_8_H_16_O	128.2	1296.1	505.919	1.40781	0.95 ± 0.07^a^	0.77 ± 0.11^a^	0.71 ± 0.04^a^
18		2-Phenyl-2-butenal	4411-89-6	C_10_H_10_O	146.2	1250.5	447.756	1.24687	0.67 ± 0.03^b^	0.36 ± 0.08^a^	0.34 ± 0.07^a^
19		3-Methyl-2-butenal	107-86-8	C_5_H_8_O	84.1	1236.3	429.703	1.35340	1.80 ± 0.01^b^	1.83 ± 0.00^b^	1.55 ± 0.07^a^
20		(Z)-4-Heptenal	6728-31-0	C_7_H_12_O	112.2	1238.7	432.749	1.14188	0.96 ± 0.06^a^	1.05 ± 0.00^a^	0.80 ± 0.12^a^
21		(E)-2-Hexen-1-al M	6728-26-3	C_6_H_10_O	98.1	1221.1	410.270	1.17731	1.56 ± 0.05^b^	0.43 ± 0.07^a^	0.37 ± 0.06^a^
22		(E)-2-Hexen-1-al D	6728-26-3	C_6_H_10_O	98.1	1221.1	410.270	1.51218	2.36 ± 0.23^b^	0.07 ± 0.01^a^	0.09 ± 0.02^a^
23		Heptaldehyde M	111-71-7	C_7_H_14_O	114.2	1193.4	375.056	1.33333	1.28 ± 0.02^c^	0.78 ± 0.10^a^	0.99 ± 0.05^b^
24		Heptaldehyde D	111-71-7	C_7_H_14_O	114.2	1193.6	375.318	1.68816	1.39 ± 0.21^c^	0.26 ± 0.07^a^	0.68 ± 0.03^b^
25		(Z)-2-Methylpent-2-enal M	623-36-9	C_6_H_10_O	98.1	1166.1	342.871	1.16212	1.65 ± 0.03^b^	1.77 ± 0.00^c^	1.53 ± 0.07^a^
26		(Z)-2-Methylpent-2-enal D	623-36-9	C_6_H_10_O	98.1	1163.6	339.920	1.49646	3.91 ± 0.05^c^	2.28 ± 0.08^a^	2.45 ± 0.07^b^
27		(E)-2-Pentenal M	1576-87-0	C_5_H_8_O	84.1	1136.2	307.694	1.10199	1.31 ± 0.06^b^	0.39 ± 0.03^a^	0.52 ± 0.10^a^
28		(E)-2-Pentenal D	1576-87-0	C_5_H_8_O	84.1	1135.6	306.971	1.35849	0.69 ± 0.11^b^	0.05 ± 0.00^a^	0.13 ± 0.03^a^
29		1-Hexanal	66-25-1	C_6_H_12_O	100.2	1099.6	264.706	1.54961	3.64 ± 0.09^c^	3.11 ± 0.27^b^	1.42 ± 0.23^a^
30		2-Methyl-2-propenal	78-85-3	C_4_H_6_O	70.1	890.4	137.248	1.04855	1.03 ± 0.06^a^	1.20 ± 0.07^b^	1.25 ± 0.02^b^
31	Alcohols	1,2-Ethanediol	107-21-1	C_2_H_6_O_2_	62.1	1613.5	929.235	1.09012	2.69 ± 0.14^c^	2.33 ± 0.15^b^	0.66 ± 0.07^a^
32		3-Furanmethanol	4412-91-3	C_5_H_6_O_2_	98.1	1679.0	1016.466	1.09156	2.48 ± 0.57^b^	2.41 ± 0.10^b^	0.66 ± 0.05^a^
33		2-Furanmethanol	98-00-0	C_5_H_6_O_2_	98.1	1657.3	987.517	1.12580	0.26 ± 0.09^a^	0.29 ± 0.02^a^	0.15 ± 0.03^a^
34		1-Octanol	111-87-5	C_8_H_18_O	130.2	1554.5	850.505	1.46718	0.18 ± 0.04^a^	0.15 ± 0.01^a^	0.12 ± 0.01^a^
35		2-Ethyl hexanol	104-76-7	C_8_H_18_O	130.2	1491.8	766.924	1.41295	0.22 ± 0.07^b^	0.09 ± 0.00^a^	0.08 ± 0.00^a^
36		1-Octen-3-ol	3391-86-4	C_8_H_16_O	128.2	1453.9	716.329	1.15416	0.25 ± 0.06^b^	0.07 ± 0.01^a^	0.05 ± 0.01^a^
37		2-Butoxyethanol	111-76-2	C_6_H_14_O_2_	118.2	1457.9	721.755	1.20376	0.93 ± 0.05^b^	0.82 ± 0.05^b^	0.26 ± 0.05^a^
38		(E)-2-Hexen-1-ol	928-95-0	C_6_H_12_O	100.2	1405.2	651.423	1.18203	0.13 ± 0.02^a^	0.13 ± 0.02^a^	0.25 ± 0.11^b^
39		2-Hexen-1-ol	2305-21-7	C_6_H_12_O	100.2	1398.5	642.450	1.52166	0.26 ± 0.10^a^	0.27 ± 0.07^a^	0.28 ± 0.04^a^
40		1-Hexanol M	111-27-3	C_6_H_14_O	102.2	1360.4	591.656	1.32372	1.35 ± 0.14^c^	1.02 ± 0.02^b^	0.34 ± 0.01^a^
41		1-Hexanol D	111-27-3	C_6_H_14_O	102.2	1358.9	589.678	1.63334	0.83 ± 0.22^b^	0.37 ± 0.01^a^	0.09 ± 0.01^a^
42		1-Hexanol T	111-27-3	C_6_H_14_O	102.2	1358.4	589.085	1.98238	0.21 ± 0.10^b^	0.06 ± 0.00^a^	0.04 ± 0.01^a^
43		(E)-3-Hexen-1-ol	928-97-2	C_6_H_12_O	100.2	1320.8	538.925	1.24184	0.85 ± 0.03^c^	0.54 ± 0.07^b^	0.10 ± 0.02^a^
44		4-Methyl pentanol	626-89-1	C_6_H_14_O	102.2	1294.3	503.620	1.61687	0.35 ± 0.07^b^	0.19 ± 0.04^a^	0.11 ± 0.02^a^
45		Cumin alcohol	536-60-7	C_10_H_14_O	150.2	1292.1	500.774	1.32394	1.68 ± 0.03^c^	1.43 ± 0.02^b^	0.62 ± 0.12^a^
46		Anisyl alcohol	105-13-5	C_8_H_10_O_2_	138.2	1272.4	475.694	1.08222	0.19 ± 0.05^a^	0.13 ± 0.01^a^	0.07 ± 0.02^a^
47		1-Pentanol M	71-41-0	C_5_H_12_O	88.1	1257.6	456.830	1.25161	1.36 ± 0.11^c^	0.77 ± 0.07^b^	0.43 ± 0.08^a^
48		1-Pentanol D	71-41-0	C_5_H_12_O	88.1	1256.9	455.923	1.51267	0.81 ± 0.09^b^	0.20 ± 0.02^a^	0.10 ± 0.03^a^
49		2-Ethoxyethanol	110-80-5	C_4_H_10_O_2_	90.1	1256.0	454.713	1.09024	1.44 ± 0.15^c^	0.41 ± 0.02^b^	0.17 ± 0.03^a^
50		2-Methyl-1-butanol D	137-32-6	C_5_H_12_O	88.1	1236.0	429.267	1.47239	3.03 ± 0.22^b^	2.98 ± 0.07^b^	2.34 ± 0.19^a^
51		2-Methyl-1-butanol M	137-32-6	C_5_H_12_O	88.1	1236.2	429.485	1.22120	0.92 ± 0.26^c^	0.70 ± 0.01^b^	0.60 ± 0.04^a^
52		Isohexyl alcohol	123-51-3	C_5_H1_2_O	88.1	1214.9	402.461	1.24665	0.34 ± 0.12^a^	0.15 ± 0.01^a^	0.24 ± 0.04^a^
53		4-Methyl-2-pentanol	108-11-2	C_6_H_14_O	102.2	1186.8	367.202	1.54890	0.69 ± 0.38^a^	2.88 ± 0.34^b^	1.54 ± 0.29^a^
54		1-Butanol	71-36-3	C_4_H_10_O	74.1	1153.7	328.224	1.17949	0.96 ± 0.14^c^	0.37 ± 0.03^a^	0.59 ± 0.03^b^
55		2-Pentanol	6032-29-7	C_5_H_12_O	88.1	1120.6	289.380	1.20304	0.08 ± 0.01^a^	0.52 ± 0.09^c^	0.30 ± 0.11^b^
56		Ethanol	64-17-5	C_2_H_6_O	46.1	939.0	158.564	1.14001	2.80 ± 0.17^b^	2.72 ± 0.08^b^	1.66 ± 0.55^a^
57		2-Octanol	123-96-6	C_8_H_18_O	130.2	940.1	159.063	1.43548	0.05 ± 0.00^a^	0.28 ± 0.04^c^	0.13 ± 0.01^b^
58	Esters	Ethyl 2-phenylacetate	101-97-3	C1_0_H_12_O_2_	164.2	1761.5	1126.511	1.77212	0.48 ± 0.10^c^	0.30 ± 0.04^b^	0.12 ± 0.04^a^
59		γ-Heptalactone	105-21-5	C_7_H_12_O_2_	128.2	1762.9	1128.376	1.25902	1.01 ± 0.06^a^	1.29 ± 0.10^c^	1.20 ± 0.23^b^
60		Ethyl 3-hydroxyhexanoate	2305-25-1	C_8_H_16_O_3_	160.2	1733.4	1089.032	1.28130	0.59 ± 0.06^a^	1.43 ± 0.09^b^	1.11 ± 0.23^b^
61		Benzyl acetate M	140-11-4	C_9_H_10_O_2_	150.2	1711.7	1060.166	1.31132	1.26 ± 0.01^b^	1.34 ± 0.01^c^	1.08 ± 0.04^a^
62		Benzyl acetate D	140-11-4	C_9_H1_0_O_2_	150.2	1712.8	1061.541	1.76153	0.37 ± 0.01^c^	0.25 ± 0.02^b^	0.14 ± 0.03^a^
63		Hexanoic acid hexyl ester	6378-65-0	C_12_H_24_O_2_	200.3	1582.9	888.346	1.60626	0.41 ± 0.15^b^	0.12 ± 0.01^a^	0.04 ± 0.01^a^
64		2-Furanmethyl acetate	623-17-6	C_7_H_8_O_3_	140.1	1518.3	802.274	1.61983	0.48 ± 0.15^b^	0.13 ± 0.03^a^	0.08 ± 0.03^a^
65		Geranyl butyrate	106-29-6	C_14_H_24_O_2_	224.3	1519.4	803.706	1.22461	0.82 ± 0.02^b^	0.84 ± 0.02^b^	0.57 ± 0.06^a^
66		3-Hydroxy-butanoic acid ethyl ester M	5405-41-4	C_6_H_12_O_3_	132.2	1500.0	777.876	1.17316	0.66 ± 0.05^c^	0.57 ± 0.01^b^	0.30 ± 0.01^a^
67		3-Hydroxy-butanoic acid ethyl ester D	5405-41-4	C_6_H_12_O_3_	132.2	1500.7	778.802	1.64825	2.00 ± 0.06^c^	1.81 ± 0.04^b^	0.60 ± 0.05^a^
68		Methyl 2-methoxybenzoate	606-45-1	C_9_H_10_O_3_	166.2	2035.9	1492.418	1.22085	0.06 ± 0.01^a^	0.26 ± 0.03^c^	0.13 ± 0.02^b^
69		2-Phenyl ethyl butanoate	103-52-6	C_12_H_16_O_2_	192.3	1436.5	693.172	1.43644	3.10 ± 0.01^b^	3.06 ± 0.03^b^	2.77 ± 0.15^a^
70		Geranyl acetate	105-87-3	C_12_H_20_O_2_	196.3	1381.4	619.729	1.22124	3.43 ± 0.55^b^	3.87 ± 0.08^b^	2.02 ± 0.28^a^
71		Linalol isobutyrate	78-35-3	C_14_H_24_O_2_	224.3	1367.3	600.925	1.22002	0.60 ± 0.07^b^	0.32 ± 0.02^a^	0.22 ± 0.06^a^
72		Hexyl propanoate	2445-76-3	C_9_H1_8_O_2_	158.2	1332.3	554.202	1.42770	0.78 ± 0.15^b^	0.09 ± 0.02^a^	0.05 ± 0.01^a^
73		Acetic acid nonyl ester	143-13-5	C_11_H_22_O_2_	186.3	1307.5	521.222	1.59047	0.20 ± 0.12^b^	0.06 ± 0.02^a^	0.06 ± 0.01^a^
74		(Z)-3-Hexen-1-yl acetate	3681-71-8	C_8_H_14_O_2_	142.2	1308.1	522.000	1.29963	0.66 ± 0.35^c^	0.34 ± 0.11^b^	0.28 ± 0.07^a^
75		Methyl enanthate	106-73-0	C_8_H_16_O_2_	144.2	1282.2	488.174	1.35489	1.28 ± 0.03^c^	0.36 ± 0.06^b^	0.04 ± 0.01^a^
76		Ethyl caproate	123-66-0	C_8_H_16_O_2_	144.2	1211.3	397.786	1.80055	0.42 ± 0.18^b^	0.40 ± 0.13^b^	0.27 ± 0.05^a^
77		2-Hydroxy-benzoic acid methyl ester	119-36-8	C_8_H_8_O_3_	152.1	1187.1	367.464	1.19979	1.29 ± 0.35^a^	1.73 ± 0.01^c^	1.52 ± 0.04^b^
78		Acetic acid heptyl ester	112-06-1	C_9_H_18_O_2_	158.2	1105.0	270.991	1.45637	0.29 ± 0.10^b^	0.05 ± 0.00^a^	0.04 ± 0.00^a^
79		Methyl 2-methylbutanoate	868-57-5	C_6_H_12_O_2_	116.2	1039.6	216.971	1.19173	0.32 ± 0.07^a^	1.22 ± 0.04^b^	1.24 ± 0.04^b^
80		Ethyl 2-methylbutanoate	7452-79-1	C_7_H_14_O_2_	130.2	1017.8	199.982	1.22932	0.39 ± 0.08^b^	0.03 ± 0.00^a^	0.13 ± 0.04^a^
81		Acetic acid hexyl ester	142-92-7	C_8_H_16_O_2_	144.2	995.7	183.417	1.41783	3.84 ± 0.03^c^	1.69 ± 0.41^a^	2.64 ± 0.10^b^
82		Acetic acid ethyl ester	141-78-6	C_4_H_8_O_2_	88.1	895.6	139.525	1.33085	0.54 ± 0.48^b^	0.62 ± 0.44^c^	0.04 ± 0.01^a^
83		1-(Acetyloxy)-2-propanone	592-20-1	C_5_H_8_O_3_	116.1	866.6	126.816	1.19224	0.25 ± 0.01^a^	0.46 ± 0.07^b^	0.42 ± 0.04^b^
84		Isobutyl propanoate	540-42-1	C_7_H_14_O_2_	130.2	862.9	125.167	1.27228	1.13 ± 0.14^a^	2.25 ± 0.01^c^	1.85 ± 0.11^b^
85		Methyl acetate D	79-20-9	C_3_H_6_O_2_	74.1	857.9	122.993	1.19058	1.23 ± 0.76^b^	2.20 ± 0.47^c^	0.83 ± 0.33^a^
86		Methyl acetate M	79-20-9	C_3_H_6_O_2_	74.1	858.1	123.080	1.02719	0.46 ± 0.16^a^	0.83 ± 0.07^b^	0.78 ± 0.11^b^
87		3-Methyl-1-butanyl acetate	123-92-2	C_7_H_14_O_2_	130.2	840.3	115.256	1.29993	1.04 ± 0.06^c^	0.84 ± 0.03^b^	0.28 ± 0.06^a^
88	Ketones	1-Phenylethanone	98-86-2	C_8_H_8_O	120.2	1635.6	958.670	1.18637	0.63 ± 0.12^b^	0.57 ± 0.07^b^	0.35 ± 0.04^a^
89		2,5-dimethyl-4-methoxy-3[2H]-furanone	4077-47-8	C_7_H_10_O_3_	142.2	1583.6	889.263	1.19221	0.69 ± 0.10^b^	0.44 ± 0.03^a^	0.30 ± 0.01^a^
90		2-Undecanone	112-12-9	C_11_H_22_O	170.3	1593.0	901.835	1.53751	0.04 ± 0.01^a^	0.13 ± 0.03^b^	0.03 ± 0.00^a^
91		2-Nonanone	821-55-6	C_9_H_18_O	142.2	1392.0	633.836	1.40166	0.15 ± 0.09^b^	0.08 ± 0.01^a^	0.05 ± 0.02^a^
92		2-Methyl-2-hepten-6-one	110-93-0	C_8_H_14_O	126.2	1341.2	566.146	1.17053	0.21 ± 0.02^b^	0.10 ± 0.02^a^	0.11 ± 0.02^a^
93		Cyclohexanone	108-94-1	C_6_H_10_O	98.1	1320.9	539.010	1.45089	0.26 ± 0.08^a^	0.15 ± 0.04^a^	0.04 ± 0.00^a^
94		1-Octen-3-one	4312-99-6	C_8_H_14_O	126.2	1307.0	520.454	1.27218	0.43 ± 0.04^b^	0.17 ± 0.05^a^	0.12 ± 0.04^a^
95		2-Methyl-3-ketotetrahydrofuran	3188-00-9	C_5_H_8_O_2_	100.1	1283.7	489.960	1.07207	0.26 ± 0.21^b^	0.34 ± 0.09^c^	0.10 ± 0.02^a^
96		3-Octanone	106-68-3	C_8_H_16_O	128.2	1266.9	468.642	1.30814	0.81 ± 0.24^c^	0.45 ± 0.04^b^	0.08 ± 0.03^a^
97		2-Octanone	111-13-7	C_8_H_16_O	128.2	1268.8	471.029	1.34984	0.32 ± 0.04^c^	0.14 ± 0.01^b^	0.05 ± 0.01^a^
98		4-Hexen-3-one	2497-21-4	C_6_H_10_O	98.1	1215.4	403.097	1.11418	0.35 ± 0.05^b^	0.22 ± 0.03^a^	0.15 ± 0.04^a^
99		2-Heptanone	110-43-0	C_7_H_14_O	114.2	1189.5	370.344	1.26274	0.48 ± 0.11^b^	0.12 ± 0.01^a^	0.10 ± 0.00^a^
100		1-Penten-3-one M	1629-58-9	C_5_H_8_O	84.1	1060.9	233.586	1.07934	0.76 ± 0.40^a^	1.90 ± 0.02^b^	0.52 ± 0.06^a^
101		1-Penten-3-one D	1629-58-9	C_5_H_8_O	84.1	1060.3	233.134	1.30392	0.12 ± 0.08^a^	2.01 ± 0.43^b^	0.12 ± 0.00^a^
102		3-Pentanone M	96-22-0	C_5_H_10_O	86.1	991.7	181.673	1.11331	0.83 ± 0.14^b^	0.87 ± 0.08^b^	0.42 ± 0.07^a^
103		3-Pentanone D	96-22-0	C_5_H_10_O	86.1	991.3	181.499	1.34947	2.32 ± 0.40^c^	1.66 ± 0.21^b^	0.47 ± 0.08^a^
104		2-Butanone	78-93-3	C_4_H_8_O	72.1	866.8	126.911	1.05275	2.04 ± 0.39^b^	0.31 ± 0.03^a^	0.31 ± 0.07^a^
105		2-Propanone	67-64-1	C_3_H_6_O	58.1	827.2	109.519	1.11768	0.51 ± 0.17^a^	0.70 ± 0.13^a^	0.35 ± 0.07^a^
106	Terpenes	Geraniol	106-24-1	C_10_H_18_O	154.3	1807.5	1187.792	1.22038	0.07 ± 0.02^a^	0.28 ± 0.06^b^	0.08 ± 0.02^a^
107		Piperitone	89-81-6	C_10_H1_6_O	152.2	1752.3	1114.303	1.28268	0.68 ± 0.04^a^	1.30 ± 0.10^b^	1.20 ± 0.23^b^
108		(−)-Carvone	99-49-0	C_10_H_14_O	150.2	1711.7	1060.166	1.82371	1.38 ± 0.16^b^	1.22 ± 0.05^b^	0.59 ± 0.04^a^
109		Citral	5392-40-5	C_10_H1_6_O	152.2	1711.7	1060.083	1.59960	0.27 ± 0.04^c^	0.18 ± 0.01^b^	0.08 ± 0.00^a^
110		β-Damascone	35044-68-9	C_13_H_20_O	192.3	1413.1	662.011	1.42430	1.84 ± 0.05^c^	1.54 ± 0.02^b^	1.19 ± 0.17^a^
111		(Z)-Jasmone	488-10-8	C_11_H_16_O	164.2	1402.8	648.193	1.32241	0.14 ± 0.03^b^	0.04 ± 0.00^a^	0.03 ± 0.00^a^
112		Theaspirane B	36431-72-8	C_13_H_22_O	194.3	1292.4	501.058	1.41960	1.34 ± 0.12^c^	0.78 ± 0.03^b^	0.31 ± 0.06^a^
113		α-Terpinolene	586-62-9	C_10_H_16_	136.2	1260.0	459.855	1.3062	0.18 ± 0.06^c^	0.13 ± 0.02^b^	0.08 ± 0.03^a^
114		3-p-Menthanol	89-78-1	C_10_H_20_O	156.3	1147.3	320.706	1.22791	0.29 ± 0.05^b^	0.05 ± 0.01^a^	0.04 ± 0.01^a^
115		β-Pinene	127-91-3	C_10_H_16_	136.2	1132.1	302.874	1.21392	0.08 ± 0.01^a^	0.41 ± 0.08^b^	0.22 ± 0.07^a^
116		cis Rose oxide	16409-43-1	C_10_H_18_O	154.3	1105.2	271.281	1.34765	0.34 ± 0.03^c^	0.12 ± 0.02^b^	0.06 ± 0.01^a^
117		α-Pinene	80-56-8	C_10_H_16_	136.2	939.8	158.897	1.21456	0.43 ± 0.09^a^	0.96 ± 0.08^b^	0.46 ± 0.03^a^
118	Pyrazines	2-Isobutyl-3-methoxypyrazine	24683-00-9	C_9_H_14_N_2_O	166.2	1503.7	782.813	1.30013	0.89 ± 0.04^c^	0.52 ± 0.02^b^	0.09 ± 0.00^a^
119		2-Acetyl-3,5-dimethylpyrazine	54300-08-2	C_8_H_10_N_2_O	150.2	1652.2	980.725	1.22395	0.15 ± 0.05^b^	0.17 ± 0.04^b^	0.09 ± 0.02^a^
120		2,3,5,6-Tetramethylpyrazine	1124-11-4	C_8_H_12_N_2_	136.2	1449.3	710.271	1.20376	0.38 ± 0.07^b^	0.15 ± 0.03^a^	0.19 ± 0.07^a^
121		2,3-Dimethyl-5-ethylpyrazine	15707-34-3	C_8_H_12_N_2_	136.2	1440.1	698.021	1.23284	1.18 ± 0.01^a^	1.26 ± 0.02^b^	1.16 ± 0.09^a^
122		Propylpyrazine	18138-03-9	C_7_H_10_N_2_	122.2	1430.4	685.005	1.21495	1.28 ± 0.12^a^	1.87 ± 0.27^c^	1.65 ± 0.41^b^
123		2-Ethyl-5-methylpyrazine	13360-64-0	C_7_H_10_N_2_	122.2	1413.1	662.011	1.6711	1.26 ± 0.02^c^	1.03 ± 0.02^b^	0.79 ± 0.13^a^
124		2,3-Dimethylpyrazine	5910-89-4	C_6_H_8_N_2_	108.1	1339.2	563.473	1.10885	0.36 ± 0.16^a^	0.54 ± 0.12^a^	0.15 ± 0.03^a^
125		2-Methylpyrazine	109-08-0	C_5_H_6_N_2_	94.1	1292.0	500.539	1.07953	0.63 ± 0.06^c^	0.42 ± 0.01^b^	0.18 ± 0.02^a^
126		2,3,5-Trimethylpyrazine	14667-55-1	C_7_H_10_N_2_	122.2	1458.0	721.841	1.62526	0.71 ± 0.13^c^	0.45 ± 0.08^b^	0.06 ± 0.01^a^
127		2-Ethyl-6-methylpyrazine	13925-03-6	C_7_H_10_N_2_	122.2	998.7	185.050	1.19568	1.09 ± 0.05^b^	1.05 ± 0.08^b^	0.89 ± 0.31^a^
128	Sulfides	Dipropyl trisulfide D	6028-61-1	C_6_H1_4_S_3_	182.4	1650.0	977.815	1.61925	2.88 ± 0.07^b^	2.65 ± 0.29^a^	2.61 ± 0.26^a^
129		Dipropyl trisulfide M	6028-61-1	C_6_H_14_S_3_	182.4	1651.7	980.160	1.33441	1.09 ± 0.02^a^	1.15 ± 0.02^b^	1.10 ± 0.06^a^
130		Bis(2-methyl-3-furanyl) disulfide	28588-75-2	C_10_H_10_O_2_S_2_	226.3	1518.8	802.877	1.40447	0.63 ± 0.04^c^	0.42 ± 0.03^b^	0.21 ± 0.04^a^
131		Allyl disulfide	2179-57-9	C_6_H_10_S_2_	146.3	1478.7	749.38	1.63977	1.51 ± 0.12^c^	1.26 ± 0.09^b^	0.28 ± 0.05^a^
132		Dipropyl disulfide	629-19-6	C_6_H1_4_S_2_	150.3	1386.3	626.299	1.46732	3.92 ± 0.03^b^	3.90 ± 0.00^b^	3.75 ± 0.05^a^
133		4,5-Dimethylthiazole	3581-91-7	C_5_H_7_NS	113.2	1361.5	593.238	1.09572	0.19 ± 0.09^c^	0.16 ± 0.03^b^	0.10 ± 0.03^a^
134		2,4-Dimethylthiazole	541-58-2	C_5_H_7_NS	113.2	1266.9	468.642	1.45547	1.30 ± 0.32^c^	0.73 ± 0.06^b^	0.17 ± 0.06^a^
135	Other categories	Isopentanoic acid	503-74-2	C_5_H_10_O_2_	102.1	1648.6	976.029	1.49073	0.61 ± 0.06^b^	0.55 ± 0.04^b^	0.24 ± 0.02^a^
136		Triethylenediamine	280-57-9	C_6_H_12_N_2_	112.2	1518.3	802.274	1.50954	1.03 ± 0.18^b^	0.47 ± 0.06^a^	0.21 ± 0.06^a^
137		Acetic acid M	64-19-7	C_2_H_4_O_2_	60.1	1443.8	702.870	1.04938	1.43 ± 0.25^a^	1.97 ± 0.05^a^	1.64 ± 0.11^a^
138		Acetic acid D	64-19-7	C_2_H_4_O_2_	60.1	1443.5	702.487	1.15901	0.51 ± 0.22^a^	1.06 ± 0.13^b^	0.23 ± 0.07^a^
139		2H-1-Benzopyran-2-one	91-64-5	C_9_H_6_O_2_	146.1	1436.1	692.661	1.67808	0.93 ± 0.00^b^	0.93 ± 0.01^b^	0.84 ± 0.03^a^
140		1,4-Dichlorobenzene	106-46-7	C_6_H_4_Cl_2_	147	1420.5	671.837	1.13267	0.20 ± 0.06^b^	0.04 ± 0.00^a^	0.04 ± 0.01^a^
141		p-Methyl anisole	104-93-8	C_8_H_10_O	122.2	1409.9	657.704	1.12316	0.09 ± 0.01^a^	0.40 ± 0.06^b^	0.06 ± 0.01^a^
142		Diphenyl ether	101-84-8	C_12_H_10_O	170.2	1405.7	652.141	1.29524	0.23 ± 0.06^b^	0.06 ± 0.00^a^	0.05 ± 0.01^a^
143		N, N-Dimethylbenzylamine	103-83-3	C_9_H_13_N	135.2	1355.3	584.932	1.22238	0.26 ± 0.09^b^	0.05 ± 0.00^a^	0.04 ± 0.01^a^
144		2-Methoxy-4-allylphenol	97-53-0	C_10_H_12_O_2_	164.2	1354.5	583.885	1.27491	0.26 ± 0.04^b^	0.05 ± 0.00^a^	0.03 ± 0.01^a^
145		2-Ethylpyridine	100-71-0	C_7_H_9_N	107.2	1306.0	519.237	1.09579	0.48 ± 0.09^b^	0.09 ± 0.01^a^	0.10 ± 0.00^a^
146		Sesamol	533-31-3	C_7_H_6_O_3_	138.1	1300.4	511.712	1.19382	0.55 ± 0.21^b^	0.46 ± 0.05^b^	0.09 ± 0.01^a^
147		Ethenylbenzene	100-42-5	C_8_H_8_	104.2	1280.6	486.080	1.76941	2.31 ± 0.07^b^	0.05 ± 0.01^a^	0.04 ± 0.00^a^
148		2,6-Dimethylpyridine	108-48-5	C_7_H_9_N	107.2	1267.3	469.105	1.07995	0.36 ± 0.07^b^	0.26 ± 0.01^b^	0.15 ± 0.02^a^
149		2-Pentyl furan	3777-69-3	C_9_H_14_O	138.2	1240.1	434.489	1.24544	1.99 ± 0.38^b^	0.82 ± 0.01^a^	0.65 ± 0.05^a^
150		Naphthalene	91-20-3	C_10_H_8_	128.2	1223.8	413.719	1.11949	0.74 ± 0.10^b^	0.23 ± 0.02^a^	0.13 ± 0.02^a^
151		4-Methylguaiacol	93-51-6	C_8_H_10_O_2_	138.2	1205.4	390.356	1.17798	0.58 ± 0.09^b^	0.05 ± 0.00^a^	0.04 ± 0.00^a^
152		1,2-Dimethylbenzene	95-47-6	C_8_H_10_	106.2	1196.1	378.509	1.07956	2.52 ± 0.28^b^	0.30 ± 0.05^a^	0.19 ± 0.02^a^
153		4-Methylphenol	106-44-5	C_7_H_8_O	108.1	1078.8	247.581	1.13300	0.56 ± 0.01^b^	0.43 ± 0.08^a^	0.31 ± 0.04^a^
154		2-Methylphenol	95-48-7	C_7_H_8_O	108.1	1056.2	229.974	1.11312	1.76 ± 0.04^a^	2.02 ± 0.02^a^	1.91 ± 0.10^a^
155		Pyrrolidine	123-75-1	C_4_H_9_N	71.1	1016.7	199.111	1.04495	2.20 ± 0.15^a^	1.72 ± 0.18^a^	1.94 ± 0.07^a^
156		2-Butylfuran	4466-24-4	C_8_H_12_O	124.2	890.1	137.133	1.17676	0.57 ± 0.04^c^	0.46 ± 0.03^b^	0.41 ± 0.21^a^
157		2-Methylbutanoic acid	116-53-0	C_5_H_10_O_2_	102.1	886.1	135.384	1.20758	0.99 ± 0.17^c^	0.79 ± 0.20^a^	0.85 ± 0.16^b^
158		1,4-Dimethylbenzene	106-42-3	C_8_H_10_	106.2	875.1	130.543	1.08013	1.13 ± 0.02^c^	0.82 ± 0.06^b^	0.45 ± 0.18^a^

The suffixes M, D, and T after a compound name represent the monomer, dimer, and trimer of the same compound, respectively. Data are presented as mean ± standard deviation (SD) (*n* = 3). Different superscript letters indicate significant differences among samples (*p* < 0.05).

**Figure 5:**
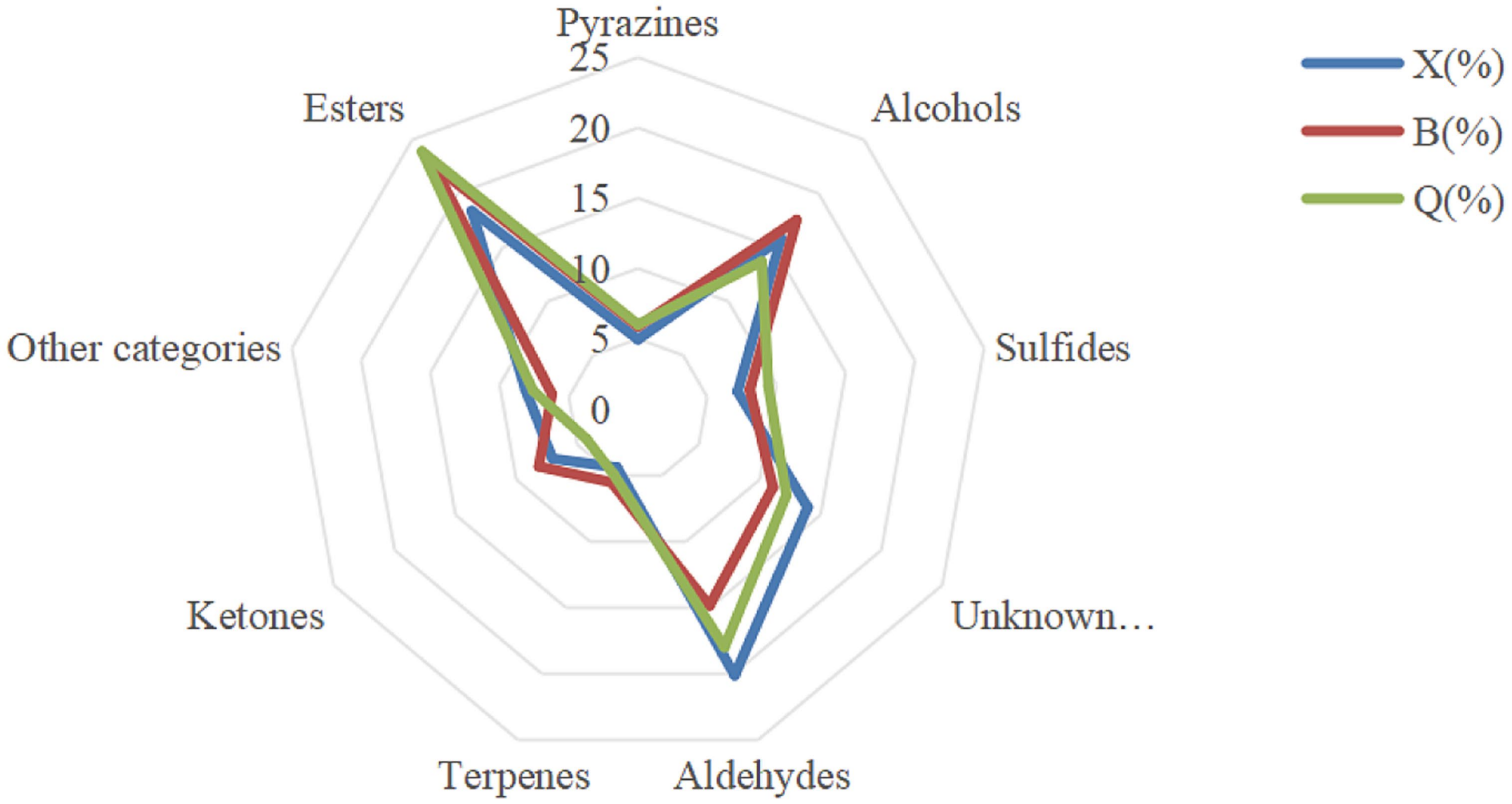
Relative percentage distribution of tentatively annotated volatile compound classes in fibrous roots, white shafts, and green leaves of *Allium schoenoprasum* L. (X: fibrous roots; B: white shafts; Q: green leaves). Values represent the relative proportions of each compound class within the total detected volatile signals of each tissue. This figure provides a descriptive class-level overview; statistical comparisons were not performed at the compound-class level unless otherwise indicated.

The relative percentage distribution of tentatively annotated volatile compound classes was further compared among the three chive tissues (Figure 5). The three tissues showed broadly similar major compound classes, including aldehydes, esters, alcohols, ketones, terpenes, pyrazines, and sulfur-containing compounds. However, differences were observed in the relative proportions of several compound classes. Aldehydes accounted for a relatively higher proportion in fibrous roots (20.12%), whereas esters were more abundant in white shafts and green leaves, accounting for 22.65 and 23.91%, respectively. Sulfur-containing compounds showed the highest relative proportion in green leaves (9.42%), followed by white shafts (8.08%) and fibrous roots (7.20%). Therefore, Figure 5 should be interpreted as a descriptive overview of class-level distribution, while tissue-dependent differences were further supported by signal-level fingerprinting, PCA, PLS–DA, and VIP analysis.

#### 3.1.3 Fingerprint analysis of volatile compounds

**Figure 6:**
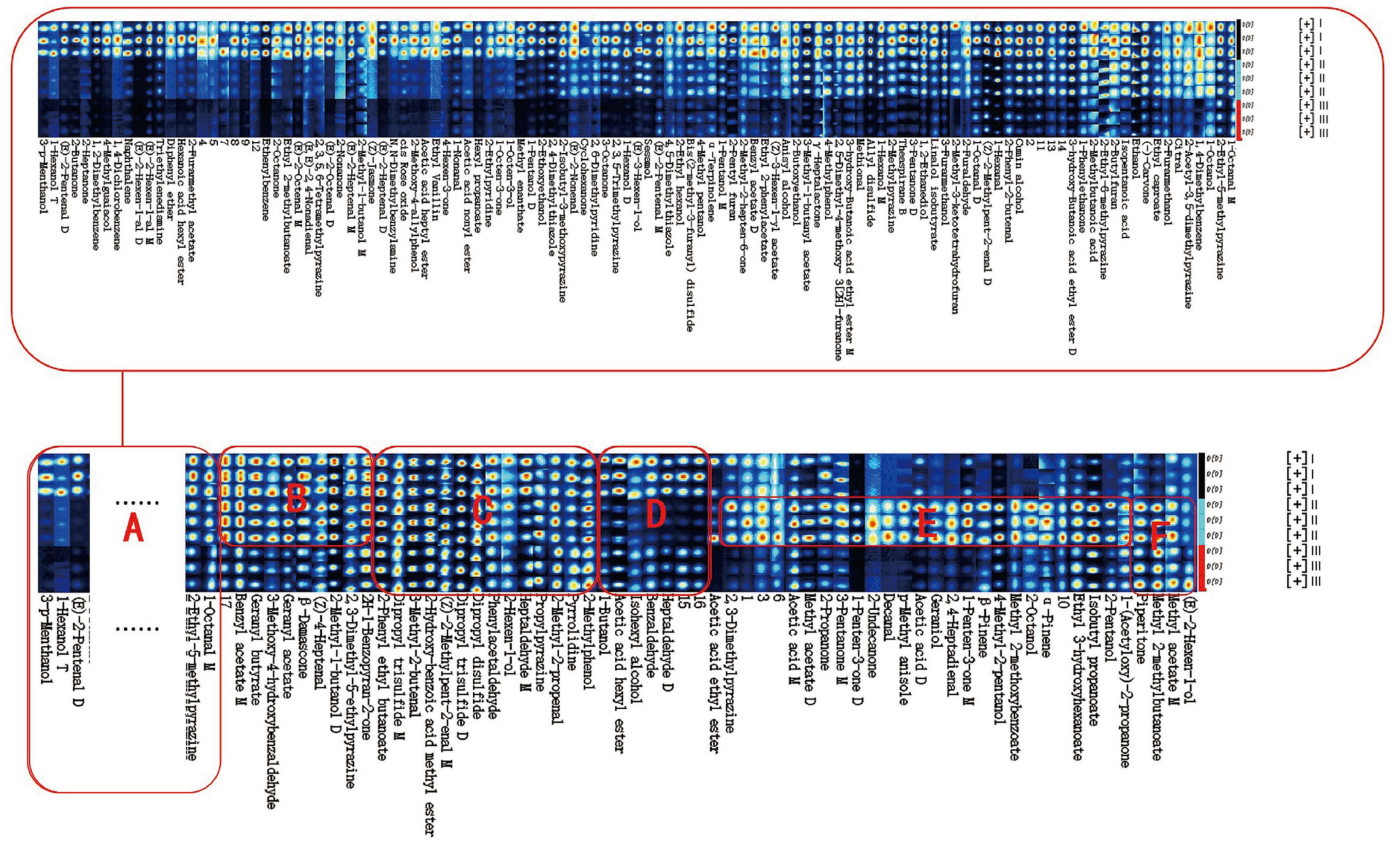
Fingerprints analysis of three types of *Allium schoenoprasum* L. by GC-IMS. (I, fibrous roots; II, white shafts; III, green leaves).

The volatile fingerprints of different chive parts were established using the Gallery module in VOCal software. The results showed clear part-dependent differences in the distribution of characteristic peaks among different regions (Figure 6). Region A mainly contained compounds with relatively high levels in the fibrous roots, including 3-p-menthanol, 1-hexanol (T), (E)-2-pentenal (D), 2-butanone, and 2-heptanone. Region B mainly consisted of compounds present at similar and relatively high levels in the fibrous roots and white shafts. Region C contained common compounds showing relatively small differences among the three parts. Region D mainly included compounds present at relatively higher levels in the fibrous roots and green leaves but lower levels in the white shafts. Region E mainly consisted of compounds enriched in the white shafts. Region F mainly contained compounds present at relatively higher levels in the white shafts and green leaves but at lower levels in the fibrous roots. These results indicate marked differences in both the composition and relative abundance of volatile compounds among the different parts of chives.

#### 3.1.4 Chemometric analysis

**Figure 7:**
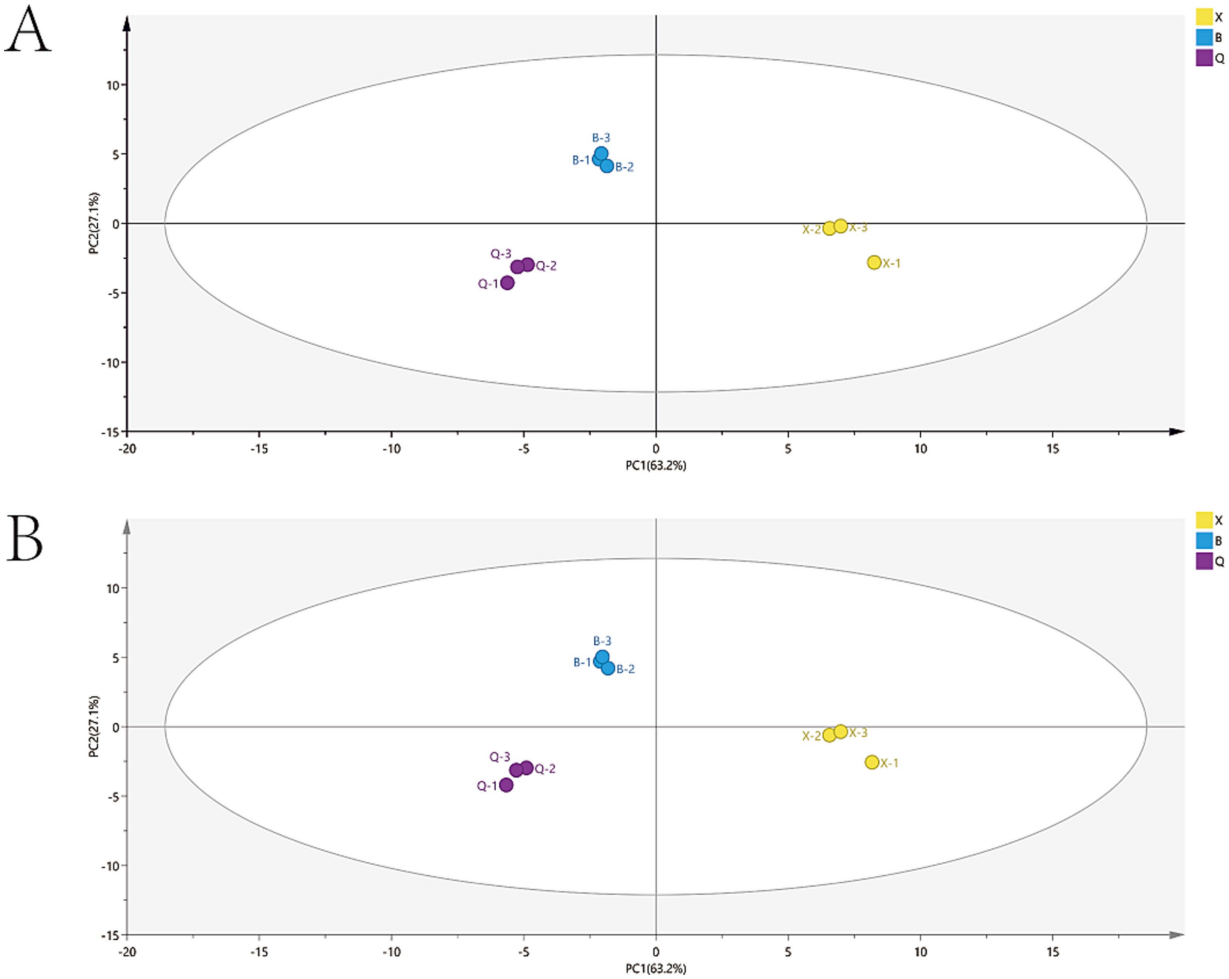
Principal component analysis of volatile compounds in three chive parts (X, fibrous roots; B, white shafts; Q, green leaves): **(A)** PCA score plot; **(B)** PLS-DA score plot.

Using SIMCA 14.1, principal component analysis (PCA) was performed on the volatile flavor compounds of chive fibrous roots, white shafts, and green leaves. As shown in Figure 7A, the cumulative variance contribution of PC1 and PC2 reached 90.3%, indicating that the model had strong explanatory power. The clear separation among fibrous roots, white shafts, and green leaves in the PCA score plot suggested substantial differences in volatile flavor composition among the three tissues. Based on the PCA results, a partial least squares-discriminant analysis (PLS-DA) model was further established (Figure 7B). The model showed excellent classification performance, with R^2^Y (cum) and Q^2^ (cum) values of 0.995 and 0.992, respectively, and an overall classification accuracy of 95%, further confirming the strong discriminatory ability of the model.

**Figure 8:**
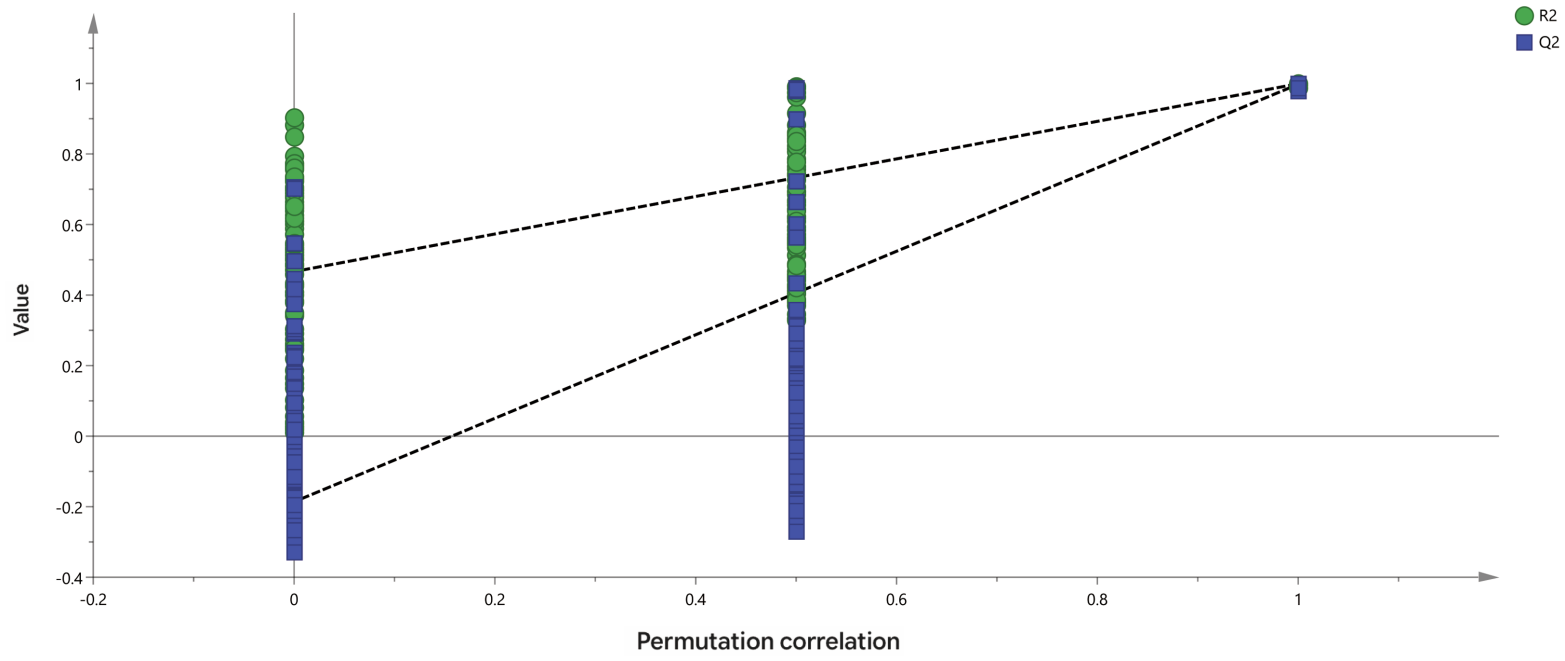
Permutation test of the PLS-DA model based on volatile compounds in three chive parts.

A permutation test (*n* = 200) was then conducted to validate the PLS-DA model, and the results are shown in Figure 8. The Q^2^ and R^2^ values of the permuted models on the left were all lower than those of the original model on the right. In addition, the regression line of Q^2^ intersected the vertical axis< 0.05, while all R^2^ values remained above zero, indicating that the established model had good predictive ability and was not overfitted.

**Figure 9:**
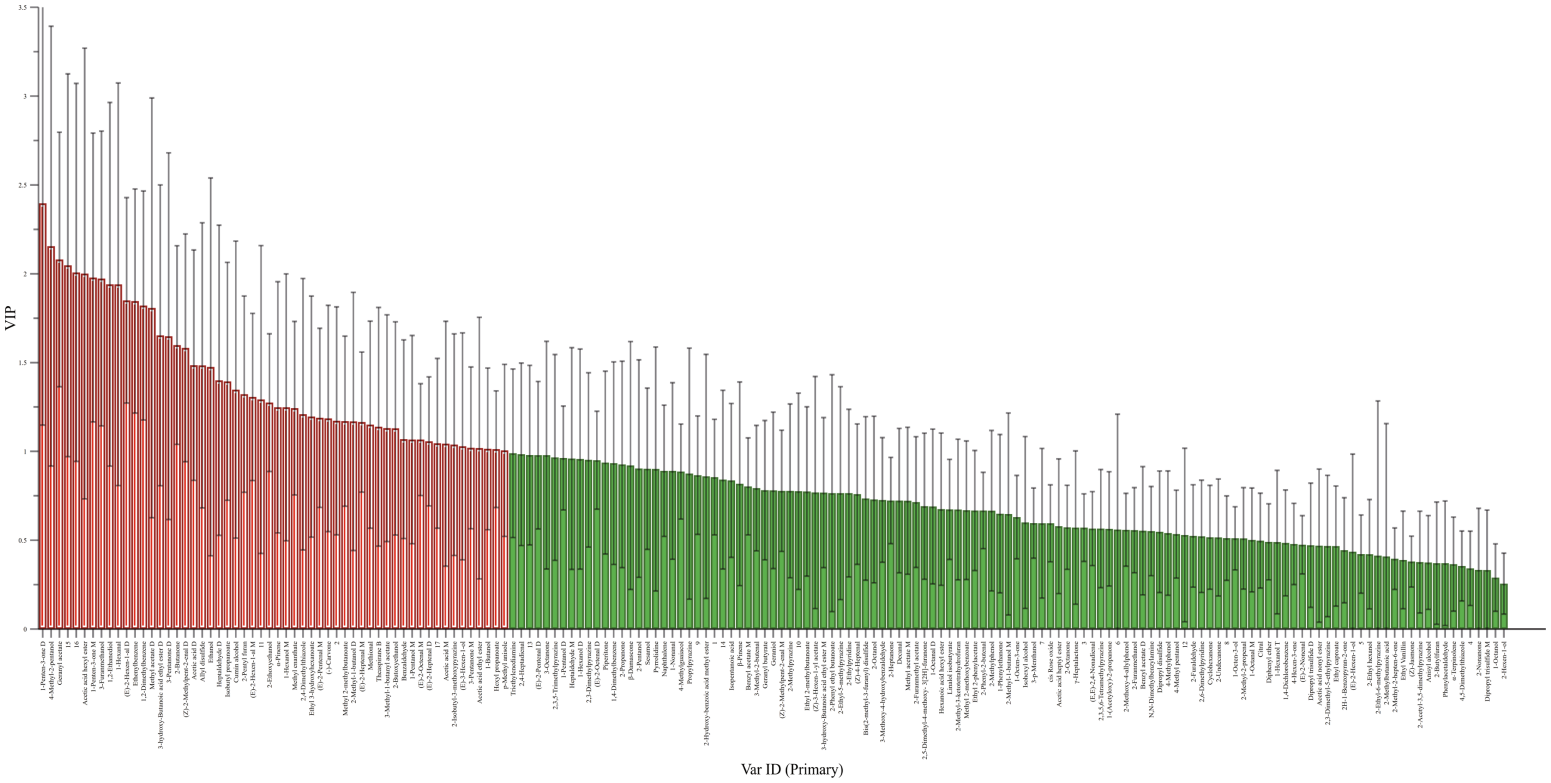
Variable importance in projection (VIP) values of volatile compounds in three chive parts.

**Figure 10:**
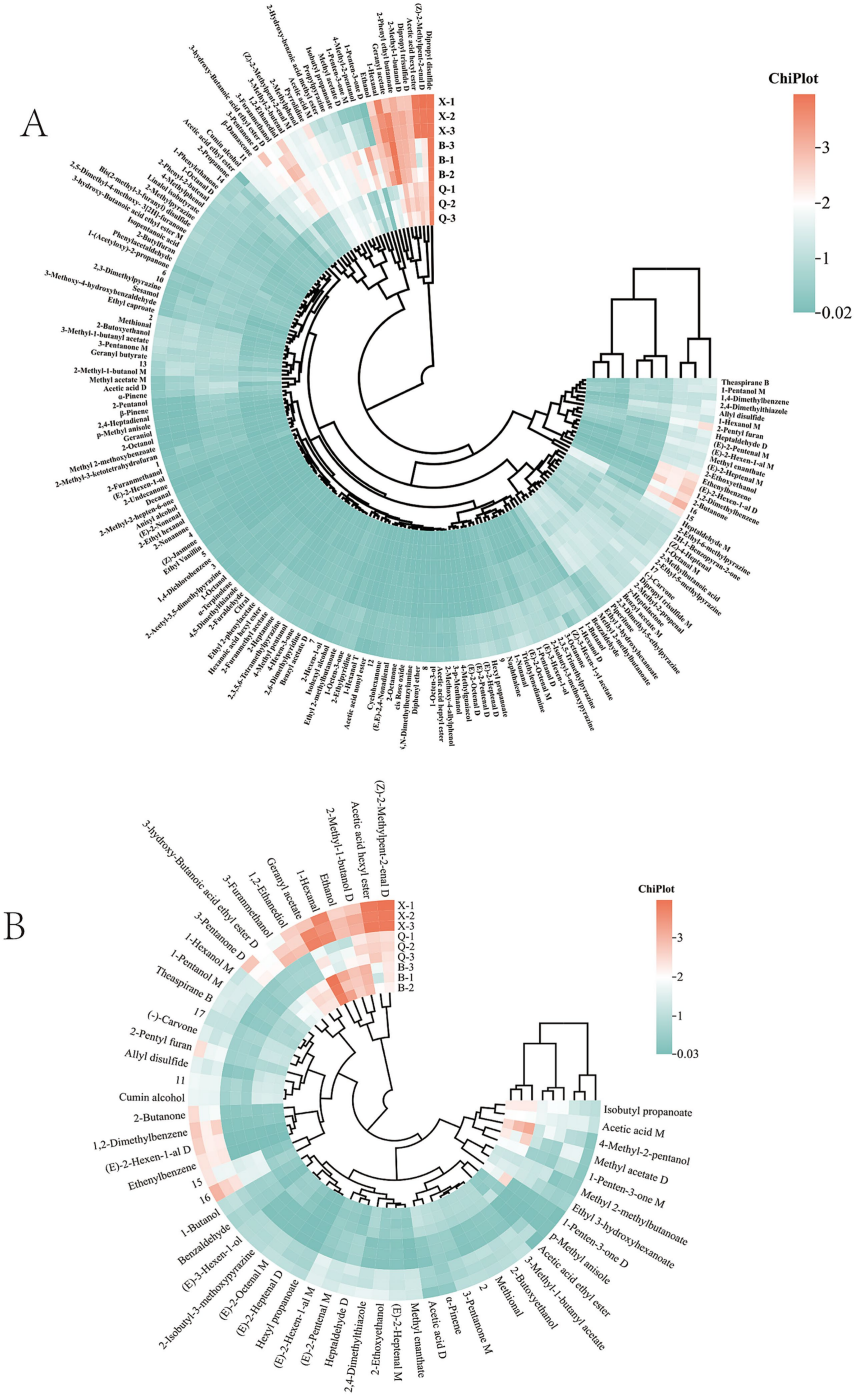
Heatmaps of volatile compounds in three chive parts (X, fibrous roots; B, white shafts; Q, green leaves): **(A)** heatmap of all volatile compounds; **(B)** heatmap of differential marker compounds.

The variable importance in projection (VIP) values derived from the PLS-DA model for the peak intensities of volatile compounds in fibrous roots, white shafts, and green leaves are shown in Figure 9. A higher VIP value indicates a greater contribution of the corresponding compound to sample discrimination. In total, 56 variables with VIP values > 1 were identified. To visualize the differences in these characteristic differential markers among fibrous roots, white shafts, and green leaves, a heatmap was constructed based on the peak volumes of the 56 selected differential markers (Figure 10B). The results showed that the heatmap generated from all 175 compounds (Figure 10A) clearly distinguished the different chive parts, while the relative abundances of the 56 differential volatile compounds also played an important role in discriminating the three sample types.

### 3.2 Radical-scavenging activities of essential oil fractions

The ABTS radical scavenging rates of all sample groups increased with increasing concentration, showing a clear dose-dependent trend. The VC group was used as the positive control. The EC50 value of the fibrous root group was 379.04 μg/mL, while those of the white shaft and green leaf groups were 459.06 μg/mL and 624.67 μg/mL, respectively.

Similarly, the DPPH radical scavenging rates of all sample groups also increased with increasing concentration, indicating a clear dose-dependent effect. The VC group served as the positive control. The EC50 value of the fibrous root group was 301.83 μg/mL, whereas the EC50 values of the white shafts and green leaf groups were 476.19 μg/mL and 671.95 μg/mL, respectively.

**Figure 11:**
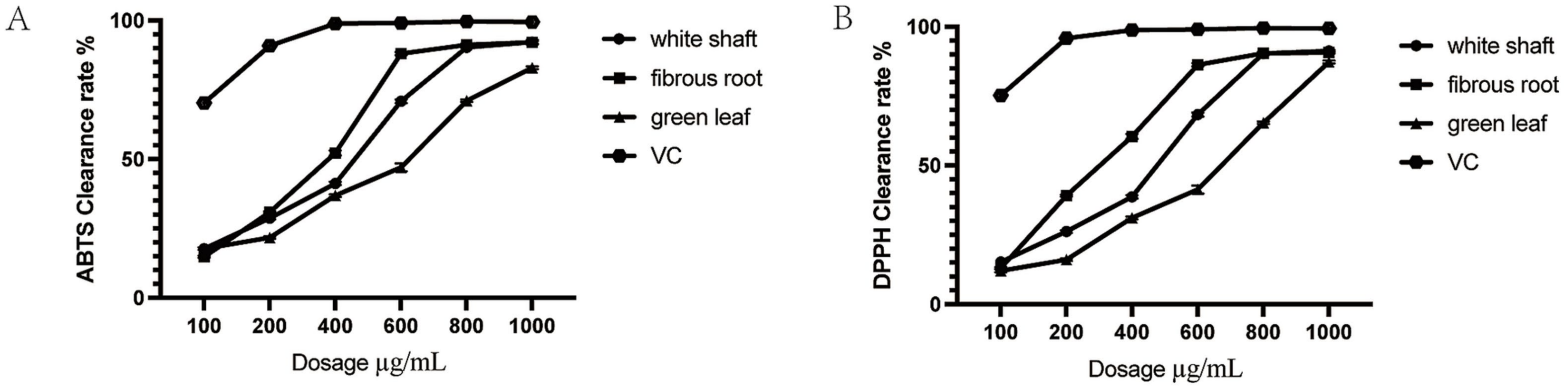
**(A)** ABTS experimental results; **(B)** DPPH experimental results.

Since a lower EC50 value indicates stronger antioxidant activity, the overall antioxidant capacity of the samples was ranked as follows: fibrous roots > white shafts > green leaves (Figure 11).

### 3.3 Cytokine-modulating effects of essential oil fractions from different chive tissues

#### 3.3.1 Effects of essential oils on RAW264.7 cell viability

**Figure 12:**
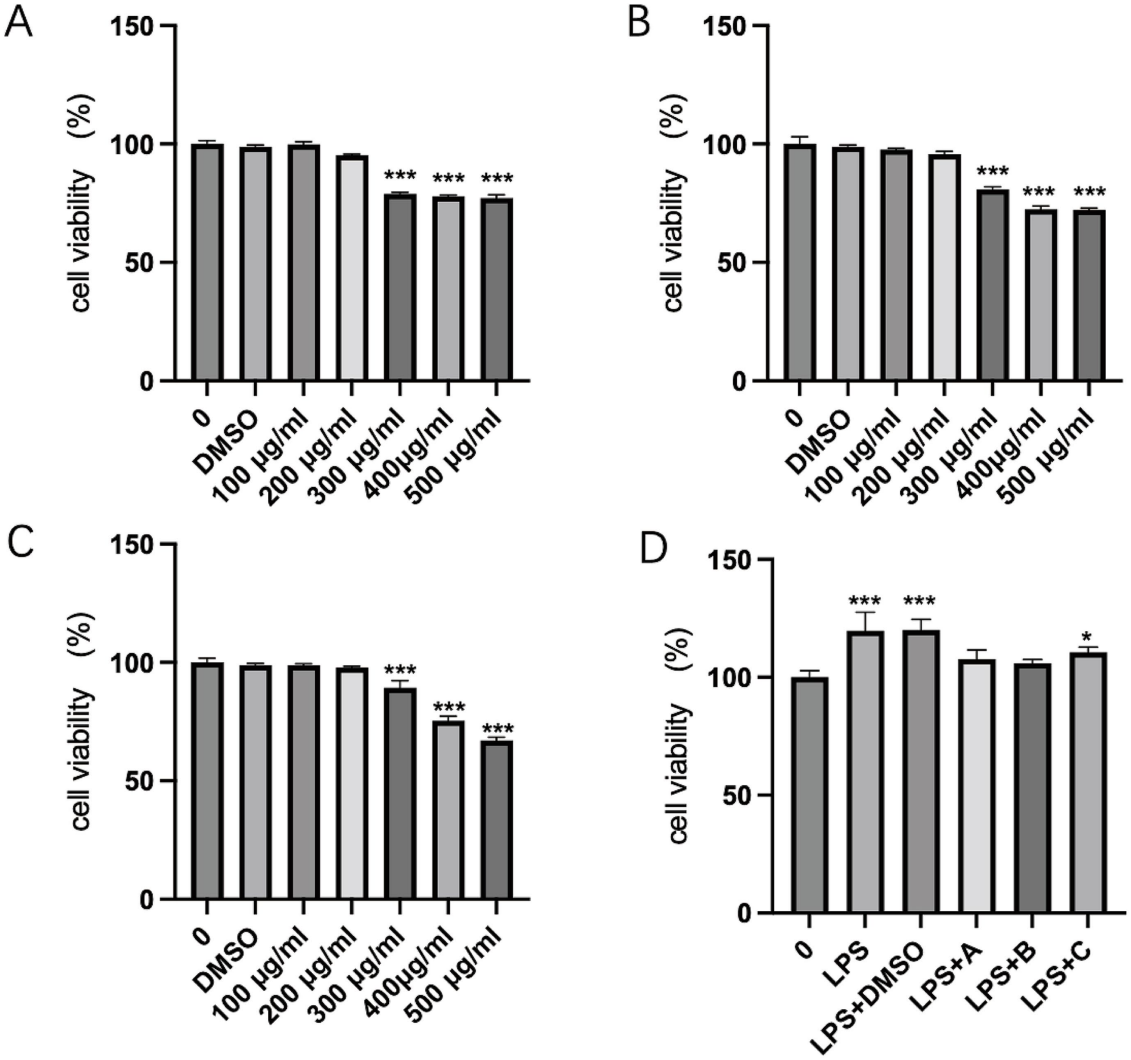
Effects of essential oils from three chive parts on the viability of RAW264.7 cells: **(A)** green leaf essential oil; **(B)** white shaft essential oil; **(C)** fibrous root essential oil; **(D)** essential oils combined with LPS. *P < 0.05, ***P < 0.0001, compared with control, *n* = 3 per group.

The effects of essential oils from different chive parts on RAW264.7 cell viability were evaluated by the CCK-8 assay. The results showed that (Figures 12A–D), at concentrations up to 200 μg/mL, essential oils from fibrous roots, white shafts, and green leaves did not significantly inhibit the viability of RAW264.7 cells, indicating that no obvious cytotoxicity was observed within this concentration range. Therefore, 200 μg/mL was selected for the subsequent cytokine assay.

#### 3.3.2 Effects of essential oils on LPS-induced inflammatory cytokine release in RAW264.7 cells

**Figure 13:**
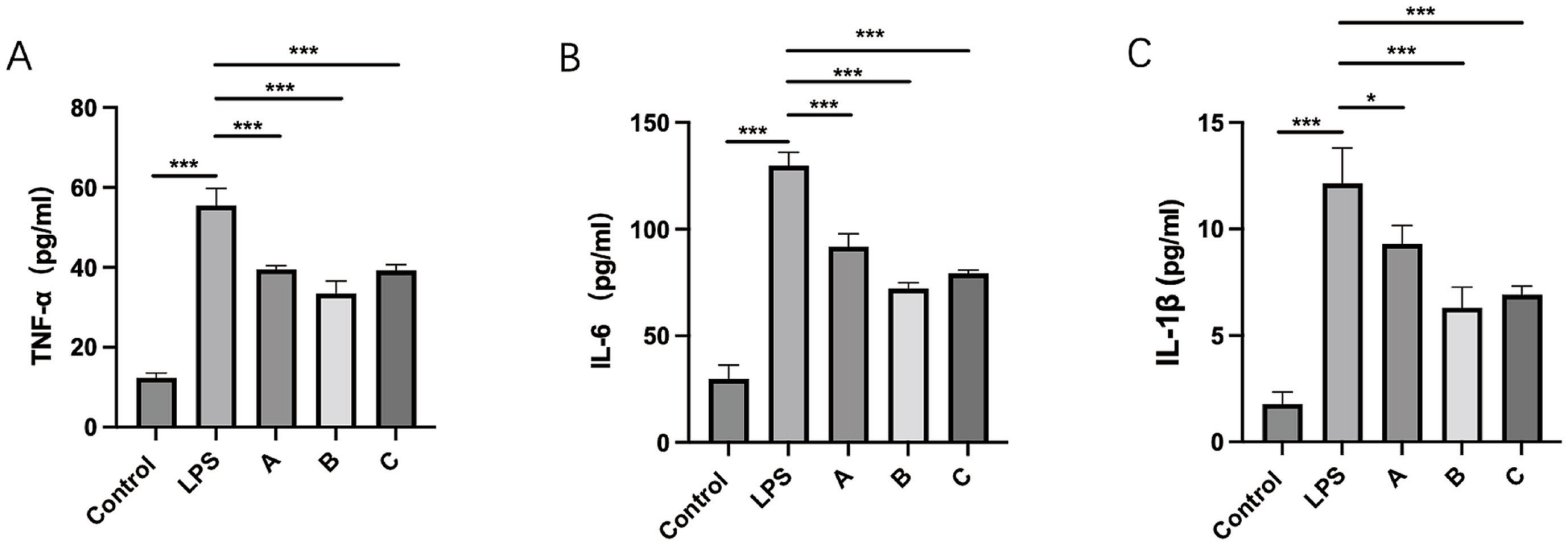
Effects of essential oils from three chive parts on LPS-induced inflammatory cytokine release in RAW264.7 cells: **(A)** TNF-α ELISA results; **(B)** IL-6 ELISA results; **(C)** IL-1β ELISA results. *P < 0.05, ***P < 0.0001, compared with control, *n* = 3 per group.

An *in vitro* inflammatory model was established by stimulating RAW264.7 cells with LPS, and the release levels of TNF-α, IL-6, and IL-1β were measured by ELISA (Figures 13A–C). Compared with the control group, the levels of TNF-α, IL-6, and IL-1β in the model group were markedly increased. Compared with the model group, essential oils from fibrous roots, white shafts, and green leaves all reduced the release of these pro-inflammatory cytokines to varying degrees. Among them, the white shaft essential oil showed the strongest inhibitory effect, followed by the fibrous root oil, while the green leaf oil showed the weakest effect.

## 4 Discussion

### 4.1 Tissue-dependent distribution of volatile compounds in different chive parts

In the present study, GC–IMS combined with chemometric analysis revealed clear tissue-dependent differences in the volatile profiles of chive fibrous roots, white shafts, and green leaves (12, 14). A total of 175 volatile signals were detected and tentatively annotated, including aldehydes, esters, alcohols, ketones, terpenes, pyrazines, sulfur-containing compounds, and other volatile categories. Because the annotation was based on GC–IMS retention information, NIST database matching, and IMS drift time data, the compound assignments should be regarded as tentative, particularly for isomeric sulfur-containing compounds, aldehydes, and terpenes. Both fingerprint analysis and multivariate models separated the three tissue types, indicating a clear tissue-dependent pattern in the volatile fingerprints of chives.

The relative abundance of major compound classes also differed among the three parts. Aldehydes were the predominant compounds in the fibrous roots, whereas esters were more abundant in the white shafts and green leaves, and sulfur-containing compounds showed the highest relative abundance in the green leaves. These findings suggest that the volatile flavor profile of chives may reflect the combined contribution of multiple volatile groups that are unevenly distributed across different anatomical parts, rather than being represented by a single tissue or compound class (11, 23, 24). Such tissue-dependent variation is plausible, given that plant organs differ in morphology, physiological function, and metabolic activity, which in turn may influence the biosynthesis, transformation, and accumulation of metabolites (25, 26).

The chemometric results further support this interpretation. The PCA model explained 90.3% of the total variance based on PC1 and PC2, while the PLS-DA model showed clear separation among the three tissue groups, with high R^2^Y (cum) and Q^2^ (cum) values. In addition, 56 compounds with VIP values >1 were screened as potential tissue-discriminating volatile markers. These VIP markers should therefore be interpreted as variables contributing to tissue discrimination rather than as confirmed bioactivity-associated compounds. Together, these results indicate that the differences among fibrous roots, white shafts, and green leaves were not limited to several individual signals, but reflected broader differences in their overall volatile fingerprints. Direct links between these discriminative volatile markers and antioxidant or anti-inflammatory activities cannot be established from the current dataset alone.

From an analytical perspective, the present results support the usefulness of GC–IMS for rapid volatile fingerprinting and discrimination of different chive tissues (27). These findings may provide a preliminary basis for evaluating tissue-associated flavor differences and for guiding further targeted compositional studies of chive-derived materials. However, GC–IMS mainly provides a rapid fingerprinting-oriented profile and has limited structural resolution for some compounds (28). Therefore, the identity and quantitative relevance of certain differential markers should be further confirmed by complementary techniques.

### 4.2 Tissue-dependent radical-scavenging activity of essential oil fractions

The antioxidant activity of the essential oil fractions from fibrous roots, white shafts, and green leaves was evaluated using ABTS and DPPH radical-scavenging assays. These two assays are commonly used to assess the direct radical-scavenging capacity of plant-derived extracts or volatile fractions under chemical reaction conditions. In the present study, all three essential oil fractions showed concentration-dependent radical-scavenging activity, indicating that the tested oils contained constituents capable of participating in free radical quenching reactions. Based on the EC50 values, the fibrous root essential oil exhibited the strongest radical-scavenging activity among the three tissues. In the ABTS assay, the EC50 values followed the order of fibrous roots, white shafts, and green leaves, indicating that the fibrous root oil had the highest ABTS radical-scavenging capacity. A similar trend was observed in the DPPH assay, where the fibrous root oil again showed the lowest EC50 value, followed by the white shaft and green leaf oils. Therefore, the antioxidant activity ranking should be interpreted as fibrous roots > white shafts > green leaves in both assay systems. This consistent pattern suggests that the radical-scavenging properties of chive essential oil fractions are tissue-dependent (29).

The stronger radical-scavenging activity of the fibrous root oil may be related to its distinct chemical characteristics at the tissue level. GC–IMS profiling showed that fibrous roots contained a broader diversity of volatile signals and a relatively higher abundance of aldehydes compared with the other tissues. Some aldehydes, alcohols, phenolic derivatives, sulfur-containing compounds, and other volatile constituents have been reported to contribute to antioxidant responses in plant-derived volatile fractions. However, the present data do not allow the antioxidant activity to be attributed to a single compound class or to specific GC–IMS markers. The antioxidant effects observed here may reflect the combined contribution of multiple components in the essential oil fractions, but this interpretation requires compositional confirmation of the tested oils (30–33).

It should also be emphasized that the GC–IMS analysis and antioxidant assays were performed on related but not identical sample preparations. GC–IMS was used to characterize the volatile fingerprints of different chive tissues, whereas the ABTS and DPPH assays were conducted using steam-distilled essential oil fractions (34). Therefore, the relationship between tissue volatile profiles and antioxidant activity should be interpreted cautiously. The current results support a tissue-associated difference in both volatile composition and radical-scavenging response, but they do not establish a direct compound–activity relationship between individual GC–IMS-detected compounds and antioxidant activity.

In addition, ABTS and DPPH assays primarily reflect chemical radical-scavenging capacity under *in vitro* conditions. These assays are useful for comparing the electron- or hydrogen-donating potential of different samples, but they do not directly represent cellular antioxidant mechanisms, bioavailability, or *in vivo* antioxidant efficacy. Thus, the present antioxidant results should be regarded as preliminary evidence of chemical radical-scavenging activity. Further studies using GC–MS confirmation of the tested essential oil fractions, cellular antioxidant assays, and activity-guided fractionation would help clarify the specific constituents and mechanisms responsible for the antioxidant responses of different chive tissues.

### 4.3 Cytokine-modulating effects of essential oil fractions in LPS-stimulated RAW264.7 cells

The cytokine-modulating effects of the essential oil fractions were evaluated using an LPS-induced RAW264.7 macrophage model. The CCK-8 assay showed that the three essential oil fractions did not markedly reduce RAW264.7 cell viability at concentrations up to 200 μg/mL, and this concentration was therefore selected for subsequent cytokine measurements. At 200 μg/mL, the three essential oil fractions reduced LPS-induced TNF-α, IL-6, and IL-1β release to varying degrees, indicating tissue-dependent cytokine-suppressive responses under the tested condition.

The cytokine-suppressive pattern did not fully parallel the radical-scavenging ranking observed in the ABTS and DPPH assays. This suggests that chemical radical-scavenging capacity and cytokine modulation should be interpreted as related but distinct biological readouts rather than as interchangeable indicators of the same activity. Therefore, the *in vitro* antioxidant data alone cannot fully explain the cytokine-suppressive responses observed in the RAW264.7 model. Conversely, cytokine inhibition at a single concentration should not be used to define the overall anti-inflammatory potency of the different essential oil fractions.

It should be emphasized that cytokine levels were measured only at 200 μg/mL. Therefore, the present data do not allow IC50 calculation, therapeutic window estimation, or rigorous potency comparison among the three essential oil fractions. In addition, because only cytokine release was measured, the upstream signaling pathways involved in this response, such as NF-κB, MAPK, or Nrf2, remain to be examined. Thus, the current results should be interpreted as preliminary evidence of cytokine-modulating activity in an LPS-stimulated macrophage model rather than as mechanistic evidence for a defined anti-inflammatory pathway.

In addition, the cytokine assay was performed using steam-distilled essential oil fractions, whereas the GC–IMS markers were obtained from tissue volatile fingerprints. Therefore, direct attribution of cytokine suppression to specific GC–IMS-detected markers is not justified. Overall, the results indicate that essential oil fractions from different chive tissues exhibit distinct *in vitro* cytokine-modulating patterns, but they do not support the conclusion that any single tissue fraction is uniformly superior across antioxidant and cytokine-related assays. Further concentration–response assays, compositional confirmation of the tested oil fractions, and pathway-level analyses would help clarify the effective concentration ranges and cellular processes involved in cytokine modulation.

### 4.4 Interpretation scope and future directions

The present study provides a comparative basis for understanding tissue-associated differences in the volatile profiles and preliminary *in vitro* bioactivity patterns of *Allium schoenoprasum* L. GC–IMS fingerprinting combined with PCA, PLS–DA, and VIP analysis showed that fibrous roots, white shafts, and green leaves possess distinct volatile characteristics. These results support a differentiated evaluation of different anatomical parts and indicate that the whole chive plant should not be regarded as a chemically uniform raw material.

When interpreting these findings, it is important to distinguish between volatile fingerprinting and bioactivity evaluation. GC–IMS was used to characterize tissue volatile profiles, whereas antioxidant and cytokine assays were conducted using steam-distilled essential oil fractions. Therefore, the observed tissue-dependent volatile markers should be interpreted as discriminative chemical features rather than direct evidence of bioactive constituents. The extraction process, including steam distillation, salting-out, solvent-assisted recovery, and solvent removal, may also influence the final composition of the tested oil fractions, particularly for reactive sulfur-containing compounds and aldehydes.

The biological assays further suggest that different chive tissues may exhibit distinct *in vitro* activity patterns. ABTS and DPPH assays reflected chemical radical-scavenging capacity, while the RAW264.7 macrophage model provided information on cytokine responses under LPS stimulation. These assays are useful for preliminary comparison, but they should not be directly extrapolated to physiological efficacy without further compositional and mechanistic confirmation. Future studies incorporating GC–MS characterization of the tested oil fractions, concentration–response cytokine assays, cellular antioxidant models, and pathway-level validation will help clarify the chemical contributors and biological relevance of tissue-specific differences in chive-derived materials.

## 5 Conclusion

This study systematically compared the volatile profiles and preliminary *in vitro* bioactivities of fibrous roots, white shafts, and green leaves of chives (*Allium schoenoprasum* L.) using GC–IMS-based volatile fingerprinting, chemometric analysis, and antioxidant and cytokine-related assays. A total of 175 volatile signals were detected and tentatively annotated. The three anatomical parts exhibited clear tissue-associated differences in volatile composition, relative abundance, and overall chemical fingerprints. Fibrous roots were characterized by a broader diversity of volatile signals and relatively abundant aldehydes, white shafts showed relatively abundant ester compounds, and green leaves contained a higher proportion of sulfur-containing compounds. PCA and PLS–DA further supported the differentiation of volatile fingerprints among the three tissue types.

The biological assays revealed distinct activity patterns among the corresponding essential oil fractions. Fibrous root essential oil showed stronger radical-scavenging activity based on EC50 values in the ABTS and DPPH assays, while cytokine-related responses in LPS-stimulated RAW264.7 macrophages varied among tissues under the tested condition. These results indicate that different parts of chives possess distinct volatile and preliminary bioactivity characteristics, suggesting that anatomical tissue type should be considered when evaluating chive-derived materials. Overall, this study provides a comparative basis for the differentiated evaluation and further targeted investigation of different chive tissues.

## Data Availability

The original contributions presented in the study are included in the article/supplementary material, further inquiries can be directed to the corresponding author.
